# Metabolomic and proteomic stratification of equine osteoarthritis

**DOI:** 10.1111/evj.14490

**Published:** 2025-02-19

**Authors:** James R. Anderson, Marie M. Phelan, Eva Caamaño‐Gutiérrez, Peter D. Clegg, Luis M. Rubio‐Martinez, Mandy J. Peffers

**Affiliations:** ^1^ Musculoskeletal and Ageing Science Institute of Life Course and Medical Sciences, University of Liverpool Liverpool UK; ^2^ Veterinary Anatomy, Physiology and Pathology Institute of Infection, Veterinary and Ecological Sciences, University of Liverpool Liverpool UK; ^3^ NMR Metabolomics Facility, Liverpool Shared Research Facilities (LivSRF) & Department of Biochemistry and Systems Biology Institute of Systems, Molecular and Integrative Biology Liverpool UK; ^4^ Computational Biology Facility, Technology Directorate & Department of Biochemistry and Systems Biology Institute of Systems, Molecular and Integrative Biology Liverpool UK; ^5^ Equine Clinical Science Institute of Infection, Veterinary and Ecological Sciences, University of Liverpool Neston UK; ^6^ Sussex Equine Hospital West Sussex UK

**Keywords:** biomarker, horse, metabolomics, osteoarthritis, proteomics, synovial fluid

## Abstract

**Background:**

Equine osteoarthritis (OA) is predominantly diagnosed through clinical examination and radiography, leading to detection only after significant joint pathology. The pathogenesis of OA remains unclear and while many medications modify the disease's inflammatory components, no curative or reversal treatments exist. Identifying differentially abundant metabolites and proteins correlated with osteoarthritis severity could improve early diagnosis, track disease progression, and evaluate responses to interventions.

**Objectives:**

To identify molecular markers of osteoarthritis severity based on histological and macroscopic grading.

**Study design:**

Cross‐sectional study.

**Methods:**

Post‐mortem synovial fluid was collected from 58 Thoroughbred racehorse joints and 83 joints from mixed breeds. Joints were histologically and macroscopically scored and categorised by OA and synovitis grade. Synovial fluid nuclear magnetic resonance metabolomic and mass spectrometry proteomic analyses were performed, individually and combined.

**Results:**

In Thoroughbreds, synovial fluid concentrations of metabolites 2‐aminobutyrate, alanine and creatine were elevated for higher OA grades, while glutamate was reduced for both Thoroughbreds and mixed breeds. In mixed breeds, concentrations of three uncharacterised proteins, lipopolysaccharide binding protein and immunoglobulin kappa constant were lower for higher OA grades; concentrations of an uncharacterised protein were higher for OA grade 1 only, and apolipoprotein A1 concentrations were higher for OA grades 1 and 2 compared with lower grades. For Thoroughbreds, gelsolin concentrations were lower for higher OA grades, and afamin was lower at a higher synovitis grade. Correlation analyses of combined metabolomics and proteomics datasets revealed 58 and 32 significant variables for Thoroughbreds and mixed breeds, respectively, with correlations from −0.48 to 0.42 and −0.44 to 0.49.

**Main Limitations:**

The study's reliance on post‐mortem assessments limits correlation with clinical osteoarthritis severity.

**Conclusions:**

Following stratification of equine OA severity through histological and macroscopic grading, synovial fluid metabolomic and proteomic profiling identified markers that may support earlier diagnosis and progression tracking. Further research is needed to correlate these markers with clinical osteoarthritis severity.

**Abbreviations**
ADAMTS: a disintegrin and metalloproteinase with thrombospondin motifs
ApoA1: apolipoprotein A1
BLAST: basic local alignment search tool
CD14: cluster of differentiation 14
COMP: cartilage oligomeric matrix protein
CPMG: Carr–Purcell–Meiboom–Gill
ECM: extracellular matrix
FDR: false discovery rate
H & E: haematoxylin and eosin
HDL: high density lipoprotein
HKJC: Hong Kong Jockey Club
LBP: lipopolysaccharide binding protein
MCP: metacarpophalangeal
MMP: matrix metalloproteinase
MSI: Metabolomics Standards Initiative
MTP: metatarsophalangeal
OA: osteoarthritis
PCA: principal component analysis
PFA: paraformaldehyde
POD: palmar/plantar osteochondral disease
PQN: probabilistic quotient normalisation
Saf O: Safranin O
SF: synovial fluid
TB: Thoroughbred
TIC: total ion current

## 1 INTRODUCTION

The age‐related degenerative musculoskeletal condition osteoarthritis (OA) is mainly characterised by articular cartilage degradation, synovitis, subchondral bone sclerosis and abnormal bone proliferation.^1^, ^2^ Although it is known that the degradation of the extracellular matrix (ECM) is driven by increased enzymatic activity of multiple matrix metalloproteinases (MMPs) and a disintegrin and metalloproteinases with thrombospondin motifs (ADAMTSs), the underlying pathogenesis of OA is yet to be fully understood.^3^, ^4^, ^5^ Currently, equine OA is predominantly diagnosed through radiography and clinical examination. However, due to the slow onset of the condition, by the time clinical signs of OA occur and a diagnosis is made, substantial damage to the joint, including articular cartilage degradation, has already occurred.^6^ There is therefore a strong need to develop accurate, OA‐specific molecular biomarkers that correlate to joint pathology. These biomarkers could improve early OA diagnosis, track disease progression, and evaluate responses to interventions as well as improve our understanding of OA pathogenesis and identify potentially novel therapeutic targets.

Currently, no equine OA‐specific biomarkers have been identified to aid an early clinical diagnosis.^7^ For human OA diagnosis, increased synovial fluid (SF) abundances of both MMPs and cartilage oligomeric matrix protein (COMP) have been identified as markers of interest.^8^ For horses, MMP activity has also shown OA diagnostic potential; however, studies investigating COMP levels have shown conflicting results, with SF COMP abundance unable to stage equine OA.^9^, ^10^, ^11^, ^12^, ^13^, ^14^, ^15^, ^16^ However, these markers are generated following significant joint pathology, including substantial articular cartilage degradation, and thus there is a need to identify markers at an earlier disease stage, when intervention would be most beneficial.

Palmar or plantar osteochondral disease (POD), located at the distal condyles of metacarpal III and metatarsal III, is a highly prevalent pathology of racehorses, resultant of repetitive joint overload during cyclic locomotion at high speed.^17^, ^18^, ^19^ POD lesions range in severity, from mild to end‐stage disease, and as the disease originates within the subchondral bone, it provides an effective model to investigate subchondral bone mediated OA.^20^

Various studies have used ^1^H nuclear magnetic resonance (NMR) to investigate metabolite markers associated with OA in various species, including pigs, dogs, humans and horses.^7^, ^21^, ^22^, ^23^, ^24^, ^25^, ^26^, ^27^, ^28^ Lacitignola et al. identified a panel of 10 metabolites which were elevated in equine OA SF compared with a normal control group.^23^ However, no studies to date have used ^1^H NMR to stratify OA to identify changes to the metabolite profile at different stages of OA severity.

Several studies have used mass spectrometry (MS) based methodologies to investigate protein changes within OA equine SF.^29^, ^30^, ^31^, ^32^, ^33^, ^34^, ^35^ Protein biomarker discovery is, however, hindered by the large protein concentration dynamic range exhibited by SF, meaning that highly abundant proteins can mask those of less abundance.^36^, ^37^ ProteoMiner™ protein enrichment columns (Bio‐Rad Laboratories Ltd.) have, however, been developed which use combinational ligand library technology, depleting highly abundant proteins and subsequently enriching low‐abundance proteins to reduce this dynamic range.^38^, ^39^, ^40^ This methodology has successfully been used to investigate OA in equine SF.^31^ However, this technique has not yet been used within SF to identify protein markers which are associated with different OA severity grades.

Increased activity of MMPs, ADAMTSs, cathepsins and serine proteases during OA leads to cartilage breakdown and the generation of OA‐specific peptide degradation products (neopeptides).^41^, ^42^, ^43^ Numerous studies investigating equine tissue have identified potential OA neopeptides of interest using ex vivo cartilage and SF.^30^, ^31^, ^41^, ^44^ OA neopeptides have also been shown to be a driver of OA pain.^45^ Therefore, identifying and quantifying neopeptide abundances, which vary in accordance with OA severity grades, could provide markers of early OA pathology and potential novel OA therapeutic and analgesic targets.^45^

It was hypothesised that interrogating SF from horses with differing severities of primary OA and those exhibiting POD pathology, as a subchondral bone mediated OA model, using both metabolomics and proteomics would identify a panel of SF molecular markers which are able to differentiate between grades of OA severity.

## 2 MATERIALS AND METHODS

### 2.1 Sample collection and processing

Post‐mortem equine samples were collected from a commercial abattoir, The Philip Leverhulme Equine Hospital, University of Liverpool and The Hong Kong Jockey Club (HKJC) Equine Hospital. Abattoir and Philip Leverhulme Equine Hospital samples were collected from horses of mixed breed and sex, representing age‐related OA disease (aged 3–35 years old) from 2014 to 2017. HKJC samples were exclusively from Thoroughbred racehorses (aged 3–10 years old) with a high prevalence of POD, providing a model for subchondral bone‐mediated OA. Samples were collected from 2005 to 2009.

### 2.2 University of Liverpool Equine Biobank (mixed breeds)

Following euthanasia at an abattoir, forelimbs were transported to the University of Liverpool. Horses euthanised at the Philip Leverhulme Equine Hospital, University of Liverpool, were processed on site. Within 8 h of euthanasia, the metacarpophalangeal (MCP) joint was opened aseptically and the distal metacarpal III photographed. SF was removed using a 10 mL syringe, transferred to a plain eppendorf tube, centrifuged at 2540*g* and 4°C for 5 min, supernatant collected, snap frozen in liquid nitrogen and stored at −80°C (Figure S1).^46^ SF was collected into separate 1 mL eppendorf tubes before snap freezing to optimise sample preservation for standardised batch analyses prior to multi ‘omics’ analysis and integration.^47^ Parasagittal wedge sections of articular cartilage/subchondral bone (measuring 4.0 cm (length) × 1.5 cm (depth) × 0.2 cm (width, articular surface)) were also sampled from the distal condylar region of metacarpal III, 1.0 cm medial to the sagittal ridge, and placed into 4% paraformaldehyde (PFA, Sigma‐Aldrich) in phosphate buffered saline (PBS, Sigma‐Aldrich) (Figure S2). Following decalcification in ethylenediaminetetraacetic acid (EDTA, Sigma‐Aldrich), these were then sectioned and stained with Haematoxylin and eosin (H & E) (TCS Biosciences Ltd) and Safranin O (BDH Chemicals).

### 2.3 Hong Kong Jockey Club (Thoroughbred racehorses)

All Thoroughbred racehorses at the HKJC were euthanised on welfare grounds for reasons unrelated to the study. Thoroughbred racehorse samples were collected using the same collection protocols as used for the University of Liverpool Equine Biobank and processed within 30 min of euthanasia. In addition, synovial membranes were also dissected, fixed in 4% PFA and processed to prepare H & E stained histology slides. For HKJC samples, both MCP and metatarsophalangeal (MTP) joints were sampled. Within the forelimb, MCP parasagittal articular cartilage/subchondral bone wedge sections were again sampled medially to the sagittal ridge. However, within the hindlimb, parasagittal sections were sampled from the lateral distal condyle of metatarsal III. This is because the medial metacarpal III condyle and lateral metatarsal III condyle sites have previously been identified as sites predisposed to pathological changes.^17^ Following snap freezing with liquid nitrogen, frozen SF samples were shipped to Liverpool on dry ice. Ages of the two sample populations were compared with Student's *t* test.

### 2.4 Osteoarthritis pathology: Macroscopic and microscopic scoring

Macroscopic and microscopic OA pathology were assessed using different scoring systems for the mixed breed and Thoroughbred racehorse cohorts. This was because Thoroughbred racehorse scores had been conducted historically, and histology slides and post‐mortem joints were subsequently unavailable to be rescored using more recently developed scoring systems. Mixed breeds' distal metacarpal III articular surfaces were assessed for macroscopic OA pathology using the equine OARSI scoring scale by two independent scorers using a previously reported scoring system.^48^ Histological sections were also assessed for microscopic OA‐related pathology using the microscopic aspect of the equine OARSI scoring scale by two independent scorers using a previously reported scoring system.^48^ For the Thoroughbred racehorse group, distal metacarpal and metatarsal III samples were assessed macroscopically and microscopically for OA‐related and POD pathology using separate previously reported scoring scales.^18^, ^49^ Marginal remodelling and dorsal impact injury categories were excluded from the macroscopic scoring scale, as these could not be scored using the joint photographs that were provided. Synovial membrane histological sections were also scored according to synovitis severity using a previously reported scoring system.^50^ Macroscopic scoring was carried out by two independent scorers, while microscopic OA and synovitis grading was carried out by one scorer only, as scoring was conducted historically and histological sections were unavailable for repeated assessment. For synovitis scoring, three separate regions of each slide were scored and then averaged.

For scoring using two independent scorers, if the final scores differed by >1, a third individual also scored the gross articular surface or histology slide, and the closest two scores were averaged. Interrater reliability was assessed using weighted Cohen's kappa coefficients, applying quadratic weighting to the observed ratings to account for the severity of disagreement between scorers.^51^, ^52^ This method was applied to the two original scores for each observation, as well as for the finalised scores used for grading, which included comparisons between an original score and the third score where appropriate.

## 3 OSTEOARTHRITIS STRATIFICATION

For mixed breeds, joints were assigned a macroscopic OA grade (maximum score = 9) as follows: grade 0 (score 0), grade 1 (score 1), grade 2 (score 2) or grade 3 (scores 3+). For microscopic OA gradings (maximum score = 20), joints were assigned as grade 0 (scores 0–1), grade 1 (scores 2–3) or grade 2 (scores of 4+).

For the Thoroughbred cohort, joints were assigned a macroscopic OA grade (maximum score = 11) as follows: grade 0 (scores 0–2), grade 1 (scores 3–4) or grade 2 (scores of 5+). For microscopic OA gradings (maximum score = 27), joints were assigned as grade 0 (scores 0–4), grade 1 (scores 3–4) or grade 2 (scores of 5+). For synovitis gradings (maximum score = 9), joints were assigned as no synovitis (scores of 0–1), low‐grade synovitis (scores of 2–4) or high‐grade synovitis (scores of 5–9).

For both the Thoroughbred racehorse and mixed breeds cohorts, microscopic OA scores were correlated to macroscopic OA scores using a Pearson correlation coefficient.^53^

## 4 ‘OMICS’ TERMINOLOGY

Within this publication there are numerous terms which are specific to ‘omics’ methodologies. Further explanation of ‘omics’ methodologies and terminologies can be found within the Equine Veterinary Journal editorial ‘Study design synopsis: “Omics” terminologies—A guide for the equine clinician’.^54^

## 5 METABOLOMICS

### 5.1 NMR sample preparation

SF was thawed over ice and centrifuged for 15 min at 13,000*g* and 4°C. 150 μL of supernatant was then diluted to produce a final volume containing 50% (v/v) SF, 40% (v/v) dd ^1^H_2_O, 100 mM PO_4_^3−^ pH 7.4 buffer (Na_2_HPO_4_, VWR International Ltd. and NaH_2_PO_4_, Sigma‐Aldrich) in deuterium oxide (^2^H_2_O, Sigma‐Aldrich) and 0.0025% (v/v) sodium azide (NaN_3_, Sigma‐Aldrich). Prepared samples were then vortexed for 1 min, centrifuged for 2 min at 13,000*g* and 4°C and, using a glass pipette, 195 μL transferred into 3 mm outer diameter NMR tubes.

### 5.2 NMR acquisition

All SF samples were individually analysed, receiving a random number to determine spectral acquisition order. A 700 MHz NMR Bruker Avance III HD spectrometer with an associated TCI cryoprobe and chilled Sample‐Jet autosampler was used to acquire all spectra. 1D ^1^H NMR spectra, using a Carr–Purcell–Meiboom–Gill (CPMG) filter to attenuate macromolecule (e.g., protein) signals, were acquired using a standard cpmgpr1d vendor pulse sequence. Spectral acquisition was carried out at 37°C with a 4 s interscan delay, 32 transients and a 15 ppm spectral width. Software programmes Topsin 3.1 and IconNMR 4.6.7 were used for acquisition and processing, carrying out automated phasing, baseline correction and a standard vendor processing routine (exponential window function with 0.3 Hz line broadening).

### 5.3 Metabolite annotation and identification

All 1D ^1^H NMR spectra were scrutinised to make sure that the minimum reporting standards were met, as outlined by the Metabolomics Society.^55^ These quality control criteria included flat baseline correction, water suppression, and consistent line widths. Spectra which did not meet these minimum requirements were removed from all subsequent analyses. Spectra were aligned to a single formate peak at 8.46 ppm. Chenomx NMR Suite 8.2 (330‐mammalian metabolite library) software was used to carry out metabolite annotations and relative abundances. Metabolite identifications were confirmed where possible using in‐house 1D ^1^H NMR metabolite spectral library standards. 1D ^1^H NMR spectra, together with annotated metabolite HMDB IDs and annotation level, are available within the MetaboLights repository (www.ebi.ac.uk/metabolights/MTBLS1645).^56^

## 6 PROTEOMICS

### 6.1 Synovial fluid processing and protein assay

SF was thawed on ice and centrifuged at 4°C at 14,000*g* for 10 min. The supernatant was treated with 1 μg/mL of hyaluronidase (bovine origin, Sigma‐Aldrich) at 37°C for 1 h, centrifuged at 1000*g* for 5 min, supernatant removed and 1 mL centrifuged through a polypropylene microcentrifuge tube filter with a 0.22 μm pore cellulose acetate membrane (Costar Spin‐X, Corning) at 5000*g* for 15 min to remove remaining insoluble particulates.^57^ A Pierce® 660 nm protein assay (Thermo Scientific) was used to determine SF protein concentrations.

### 6.2 ProteoMiner™ column processing

2 mg of protein was loaded onto ProteoMiner™ Small Capacity bead columns (Bio‐Rad Laboratories Ltd.) to achieve peptide‐based depletion of the most abundant proteins. SF samples were rotated for 2 h at room temperature and centrifuged at 1000*g* for 1 min. ProteoMiner™ beads were then washed in PBS, rotated for 5 min and centrifuged at 1000*g* for 1 min. The wash step was repeated two further times.

### 6.3 Protein digestion

To assess both high and low abundance synovial proteins, both native and ProteoMiner™ processed SF were analysed. For native SF, 100 μg of protein was used for each protein trypsin digestion. 25 mM ammonium bicarbonate (Fluka Chemicals Ltd.) containing 0.05% (w/v) RapiGest (Waters) was added to both native SF and peptide bound ProteoMiner™ beads to produce a final volume of 160 μL and heated at 80°C for 10 min. DL‐Dithiothreitol (Sigma‐Aldrich) was added (3 mM final concentration) and incubated at 60°C for 10 min. Iodoacetamide (Sigma‐Aldrich) was added (9 mM final concentration) and incubated at room temperature for 30 min in the dark. ProteoMiner™ processed samples then underwent an additional step which entailed the addition of 2 μg of Lys‐C endopeptidase (FUJIFILM Wako Pure Chemical) and incubation at 37°C for 4 h.^46^ 2 μg of proteomics grade trypsin (Sigma‐Aldrich) was added to all samples and rotated for 16 h at 37°C, followed by a second trypsin supplementation for 2 h incubation. Digests were centrifuged at 1000*g* for 1 min, the supernatant collected, trifluoroacetic acid (TFA, Sigma‐Aldrich) added (0.5% (v/v) final concentration) and rotated for 30 min at 37°C. Digests were then centrifuged at 13000*g* for 15 min at 4°C and the supernatant collected and stored at −80°C.

### 6.4 Sample processing for neopeptide analysis

After 4 h of the 16 h tryptic digestion of ProteoMiner™ processed samples, 10 μL was removed and supplemented with TFA and stored at −80°C using the same protocol as mentioned above.

### 6.5 Label free LC–MS/MS

SF digest samples were ordered by their randomised number and individually analysed using liquid chromatography tandem mass spectrometry (LC–MS/MS) on an UltiMate 3000 Nano LC System (Dionex/Thermo Scientific) coupled to a Q Exactive™ Quadrupole‐Orbitrap instrument (Thermo Scientific). Full LC–MS/MS instrument methods are described in Methods S1 and Figure S1. Tryptic peptides, which were equivalent to 200 ng of protein, were loaded onto the column and run over 60 min, 90 min and 120 min LC gradients for 4 h Lys‐C + 4 h tryptic digest ProteoMiner™ samples, native SF and 4 h Lys‐C + 16 h + 2 h tryptic digest ProteoMiner™ processed samples, respectively. Mass spectrometry proteomics data have been deposited to the ProteomeXchange Consortium via the PRIDE partner repository with the dataset identifier PXD019842 and 10.6019/PXD019842.^58^ Representative ion chromatograms are shown in Figure S3.

### 6.6 LC–MS/MS spectra processing and protein identification

Progenesis™ QI 2.0 (Nonlinear Dynamics, Waters) software was used to process raw spectral files and undertake spectral alignment, peak picking, total protein abundance normalisation and peptide/protein quantification. The 10 most intense fragment ion spectra for each feature were then exported, with peptide and protein identifications carried out with PEAKS® Studio 8.0 (Bioinformatics Solutions Inc.) using the reviewed *Equus caballus* database. Search parameters included: precursor mass error tolerance, 10.0 ppm; fragment mass error tolerance, 0.01 Da; precursor mass search type, monoisotopic; enzyme, trypsin; maximum missed cleavages, 1; non‐specific cleavage, none; fixed modifications, carbamidomethylation; variable modifications, oxidation or hydroxylation and oxidation (methionine). The false discovery rate (FDR) was set to 1%, with only proteins identified with >2 unique peptides used for quantitation.

### 6.7 Semi‐tryptic peptide identification

A ‘semi‐tryptic’ search was undertaken to identify potential synovial neopeptides. PEAKS® search parameters were kept the same as those used for protein identifications, apart from ‘Non‐specific Cleavage’ which was changed from ‘none’ to ‘one’. The exported ‘peptide ion measurements’ file from Progenesis™ was then analysed using an online neopeptide analyser software tool to identify only semi‐tryptic peptides and perform normalisation.^59^

### 6.8 Batch corrections

ProteoMiner™ processed samples for both the mixed breeds and Thoroughbred racehorse sample sets were run in three separate batches on the Q Exactive™ for protein analysis. To eliminate batch effects on the final analysis, a COMBAT batch correction was applied using MetaboAnalyst 4.0 (http://www.metaboanalyst.ca) (Figure S4).^60^ Metabolomic spectra were also acquired over three batches for both mixed breeds and Thoroughbred racehorse sample cohorts, and data also underwent COMBAT batch correction.

### 6.9 Statistical analysis of individual metabolomics and proteomics datasets

SF metabolite abundances underwent median normalisation (MetaboAnalyst 4.0) and protein abundances were normalised to the total ion current (TIC) (Progenesis™ QI 2.0). Before multivariate analysis, both metabolite and protein datasets underwent Pareto scaling.^61^ All principal component analyses (PCA), ANOVA and *t*‐tests of metabolite, protein and neopeptide abundances were conducted using MetaboAnalyst 4.0. For protein analysis, an additional filter of >2‐fold abundance was implemented. A *p* value of <0.05 was considered statistically significant following correction for multiple testing using the Benjamini‐Hochberg FDR method.^62^ All box plots were produced using SPSS 24.

### 6.10 Statistical analysis of combined metabolomics and proteomics datasets

Metabolomic and proteomic datasets were combined for the Thoroughbred racehorse cohort and combined for the mixed breeds cohort separately. Only datasets for horses which had both metabolite and protein abundance values were used for integration. Proteomics datasets were normalised to the TIC while NMR datasets were normalised via probabilistic quotient normalisation (PQN).^63^ When combining ProteoMiner™ processed SF and native SF protein abundances for the same SF sample, in which the same protein had been identified in both datasets, the higher abundance was included for analysis. The Mahalanobis distance on principal components was calculated and Chi‐squared testing was undertaken to identify potential outliers (adjusted *p* value <0.05). When combining metabolite and protein variables, categorisation was carried out in accordance with the macroscopic OA scores opposed to macroscopic OA grades (McIlwraith et al.^48^ scoring system for the mixed breeds cohort and Barr et al.^18^ for the Thoroughbred racehorse cohort). Correlations of all variables (proteins and metabolites) with macroscopic OA score were calculated using the Spearman coefficient, with significant variables after correcting for FDR (adjusted *p* value <0.05) included for further analysis. All integration analyses were undertaken using analytical routines within the software R.^64^

### 6.11 Uncharacterised proteins

Within this study, proteins which were considered uncharacterised were also analysed using BLAST (Basic local alignment search tool) to assess the similarity of their amino acid sequences to characterised proteins within the *Equus caballus* database as well as other species.^65^ Search parameters included: Matrix, blosum62; threshold, 0.1 E; filtering, none; gapped, true.

## 7 RESULTS

### 7.1 Stratification grades

Examples of microscopic OA and macroscopic OA scores are shown in Figures S5 and S6. Interrater reliability assessment between the two initial scorers for microscopic OA and macroscopic OA pathology scores of mixed breeds samples and macroscopic OA pathology scores of Thoroughbred samples identified moderate, good and moderate agreement with weighted Cohen's kappa coefficients of 0.44, 0.64, and 0.54, respectively. For the finalised scores, which included adjudicated scores via a third scorer, agreement for mixed breeds microscopic OA, mixed breeds macroscopic OA and Thoroughbred macroscopic OA scores were found to be very good, very good and good with coefficients of 0.91, 0.85 and 0.80, respectively. Histological microscopic OA scores vs. macroscopic OA scores for mixed breeds and Thoroughbred racehorse samples both showed a weak positive correlation with R^2^ coefficient values of 0.12 and 0.17, respectively (Figure S7). Joints were assigned into severity grades according to microscopic OA pathology, macroscopic OA pathology and synovitis scores (Table 1). For the Thoroughbred racehorse cohort, macroscopic OA scores were on average 30% of the highest possible score, compared with 14% for mixed breeds and microscopic scores 31% compared with 15% for mixed breeds. All scoring is shown in Tables [Link], [Link]. Overall, the Thoroughbred racehorse dataset was a younger cohort (*p* < 0.001) than the mixed breeds dataset, with average ages of 6.6 ± 1.9 years and 14.3 ± 7.6 years, respectively.

**TABLE 1:** Stratification grades according to microscopic osteoarthritis, macroscopic osteoarthritis and synovitis pathology used for synovial fluid LC–MS/MS proteomic and NMR metabolomic analysis.

Mixed breeds							
	OARSI microscopic OA grade			OARSI macroscopic OA grade			
	0	1	2	0	1	2	3
Metabolomics							
Number of joints	8	34	28	14	27	21	14
Joint types	8 × MCP	34 × MCP	28 × MCP	14 × MCP	27 × MCP	21 × MCP	14 × MCP
Age (years)	8	15	17	9	15	16	19
Sex (M/F)	1 F, 7 Unknown	6 M, 10 F, 18 Unknown	12 M, 10 F, 6 Unknown	2 M, 5 F, 7 Unknown	7 M, 12 F, 8 Unknown	9 M, 5 F, 7 Unknown	6 M, 3 F, 5 Unknown
Proteomics							
Number of joints	8	34	28	11	25	18	12
Joint types	8 × MCP	34 × MCP	28 × MCP	11 × MCP	25 × MCP	18 × MCP	12 × MCP
Age (years)	8	15	17	9	15	16	20
Sex (M/F)	1 F, 7 Unknown	6 M, 10 F, 18 Unknown	12 M, 10 F, 6 Unknown	1 M, 3 F, 7 Unknown	6 M, 11 F, 8 Unknown	6 M, 5 F, 7 Unknown	5 M, 2 F, 5 Unknown
Protein concentration (mg/mL)	5	5	4	5	4	5	6

Thoroughbred racehorses									
	Microscopic OA grade			Macroscopic OA grade			Synovitis grade		
	0	1	2	0	1	2	0	1	2
Metabolomics									
Number of joints	17	20	13	13	25	12	1	38	17
Joint types	10 × MCP, 7 × MTP	8 × MCP, 12 × MTP	9 × MCP, 4 × MTP	5 × MCP, 8 × MTP	13 × MCP, 12 × MTP	11 × MCP, 11 × MTP	1 × MTP	22 × MCP, 16 × MTP	10 × MCP, 7 × MTP
Age (years)	6	7	7	5	7	7	6	7	7
Sex (M/F)	Unknown			Unknown			Unknown		
Proteomics									
Number of joints	16	20	13	13	24	12	1	34	19
Joint types	9 × MCP, 7 × MTP	8 × MCP, 12 × MTP	9 × MCP, 4 × MTP	5 × MCP, 8 × MTP	12 × MCP, 12 × MTP	11 × MCP, 11 × MTP	1 × MTP	18 × MCP, 16 × MTP	10 × MCP, 9 × MTP
Age (years)	6	7	7	5	7	7	6	7	7
Sex (M/F)	Unknown			Unknown			Unknown		
Protein concentration (mg/mL)	10	9	13	7	9	17	16	9	12

Abbreviations: F, female; M, male; MCP, metacarpophalangeal joint; MTP, metatarsophalangeal joint; OA, osteoarthritis.

### 7.2 Palmar or plantar osteochondral disease (POD) prevalence

Within the Thoroughbred racehorse cohort, 66% (33/50) of scored joints were identified as having POD pathology present. Of those joints with POD pathology present, 58% were scored at grade 1, 30% at grade 2 and 12% at grade 3.

### 7.3 NMR metabolomics

**FIGURE 1:**
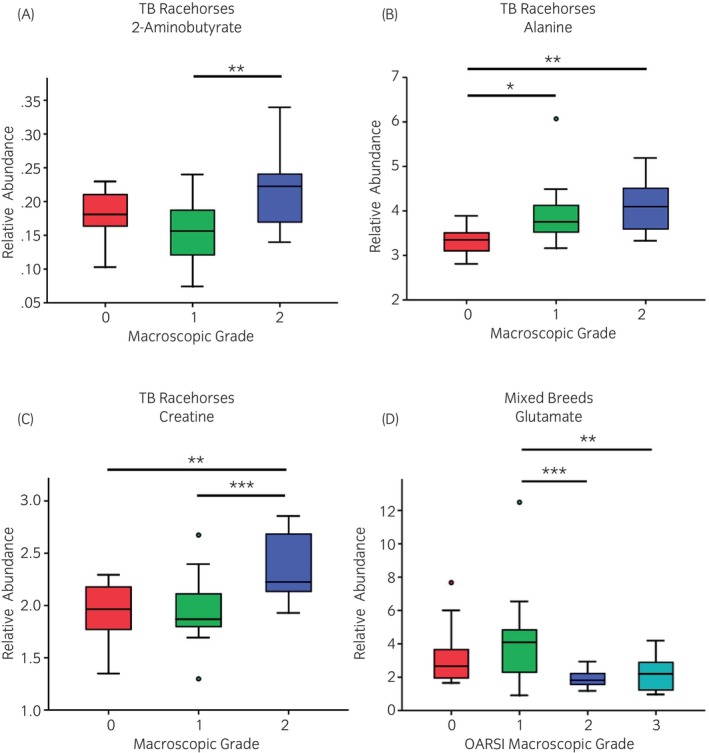
Differentially abundant metabolites within Thoroughbred (TB) racehorses (A–C) and mixed breeds (D) equine synovial fluid, categorised by macroscopic osteoarthritis (*n* = 76) grade. Thoroughbred racehorses, *n* = 50; Mixed breeds, *n* = 76. ANOVA: **p < 0.01 and ***p < 0.001.

Overall, both the mixed breeds and Thoroughbred racehorse SF 1D ^1^H NMR spectra produced similar profiles, although quantile plots revealed the mixed breeds cohort exhibited more variation between samples (Figure S8). In total, 40 metabolites were identified within equine SF (Table 2). For the mixed breeds cohort, unsupervised multivariate PCA did not identify separation between OA grades (Figure S9). However, when stratified according to macroscopic grade, glutamate levels were found to be differentially abundant, with lower concentrations identified at grades 2 and 3 compared with grade 1 (Figure 1D). PCA did not identify clear separation between severity grades of OA or synovitis for Thoroughbred racehorse SF samples (Figure S10). When stratified according to macroscopic OA pathology, three metabolites were found to be differentially abundant (Figure 1A–C). 2‐aminobutyrate concentrations were higher for grade 2 compared with grade 1 and alanine and creatine concentrations were higher for grades 1 and 2 compared with grade 0.

**TABLE 2:** Synovial fluid metabolites identified using Chenomx software.

Database identifier	Metabolite identification	Reliability
HMDB00650	2‐Aminobutyrate	MS Level 2
HMDB00357	3‐Hydroxybutyrate	MS Level 2
HMDB00754	3‐Hydroxyisovalerate	MS Level 2
HMDB01149	5‐Aminolevulinate	MS Level 2
HMDB00042	Acetate	MS Level 1
HMDB00194	Anserine	MS Level 2
HMDB00043	Betaine	MS Level 1
HMDB00097	Choline	MS Level 1
HMDB00094	Citrate	MS Level 1
HMDB00064	Creatine	MS Level 1
HMDB01511	Creatine phosphate	MS Level 2
HMDB00562	Creatinine	MS Level 1
HMDB00122	D‐Glucose	MS Level 1
HMDB04983	Dimethyl sulfone	MS Level 2
HMDB00142	Formate	MS Level 2
HMDB00123	Glycine	MS Level 1
HMDB00128	Guanidoacetate	MS Level 2
HMDB00172	Isoleucine	MS Level 1
HMDB00190	Lactate	MS Level 1
HMDB00161	L‐Alanine	MS Level 1
HMDB00062	L‐Carnitine	MS Level 2
HMDB00174	L‐Fucose	MS Level 2
HMDB00148	L‐Glutamate	MS Level 1
HMDB00641	L‐Glutamine	MS Level 1
HMDB00177	L‐Histidine	MS Level 1
HMDB00687	L‐Leucine	MS Level 1
HMDB00159	L‐Phenylalanine	MS Level 1
HMDB00158	L‐Tyrosine	MS Level 1
HMDB00883	L‐Valine	MS Level 1
HMDB00691	Malonate	MS Level 2
HMDB01844	Methylsuccinate	MS Level 2
HMDB31419	N‐Nitrosodimethylamine	MS Level 2
HMDB00895	O‐Acetylcholine	MS Level 2
HMDB00243	Pyruvate	MS Level 1
HMDB00254	Succinate	MS Level 1
HMDB00294	Urea	MS Level 1
HMDB00296	Uridine	MS Level 1
HMDB00292	Xanthine	MS Level 2
HMDB00001	π‐Methylhistidine	MS Level 2
HMDB00001	τ‐Methylhistidine	MS Level 2

*Note*: Metabolites that had also undergone identification using a 1D ^1^H NMR in‐house spectral library were assigned to Metabolomics Standards Initiative (MSI) level 1.^105^ Metabolomics Standards Initiative definitions: MS Level 1 = Identified metabolite using two or more orthogonal properties of an authentic chemical standard analysed in the researcher's laboratory. MS Level 2 = Putatively annotated metabolite which does not require matching to data for authentic chemical standards acquired within the same laboratory.

### 7.4 LC–MS/MS proteomics

Within the mixed breeds cohort, 74 native SF samples were analysed, with 68 of these additionally processed using ProteoMiner™ columns. For the Thoroughbred racehorse cohort, 56 native SF samples were analysed, with 55 of these additionally having also undergone ProteoMiner™ processing. In total, across all samples, 1834 proteins were identified. A combination of an increase in LC gradient length and ProteoMiner™ processing resulted in a 168% increase in the overall number of identified proteins compared with native SF analysis.

**FIGURE 2:**
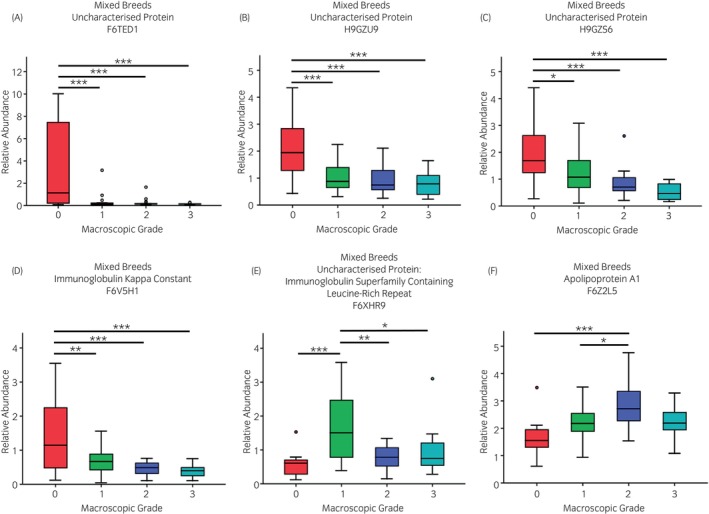
Differentially expressed proteins within native equine synovial fluid from mixed breeds when categorised by macroscopic osteoarthritis grade (*n* = 66). ANOVA: *p < 0.05; **p < 0.01; ***p < 0.001.

**FIGURE 3:**
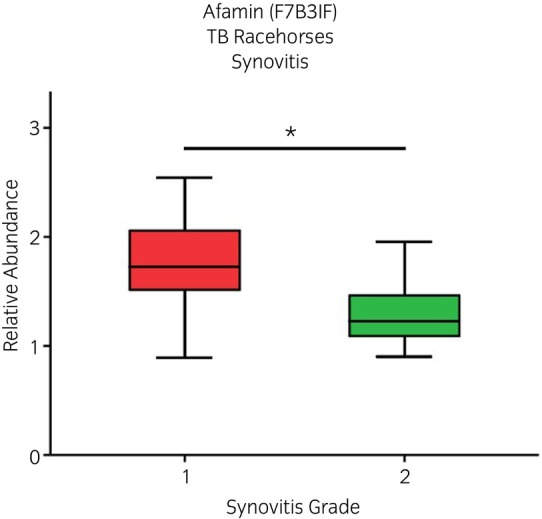
(D) Differential expression of afamin identified between synovitis grade 1 and 2 for the Thoroughbred (TB) racehorse native equine synovial fluid dataset using LC–MS/MS. *N* = 53. *t*‐test: *p < 0.05.

**FIGURE 4:**
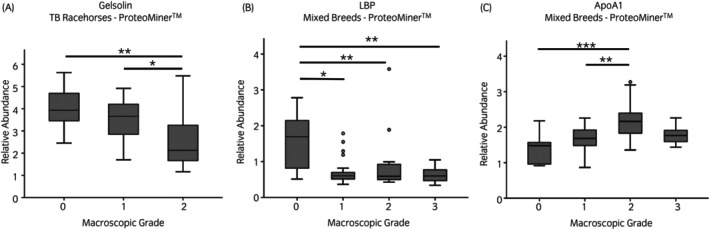
Abundances of gelsolin, lipopolysaccharide binding protein (LBP) and apolipoprotein A1 (ApoA1) within equine synovial fluid via LC–MS/MS according to macroscopic osteoarthritis grade. Thoroughbred (TB) racehorses, *n* = 47; Mixed Breeds, *n* = 60; ANOVA: *p < 0.05; **p < 0.01; ***p < 0.001.

Following PCA analyses, two Thoroughbred racehorse native SF samples were identified as outliers and removed from further analyses. For mixed breeds and Thoroughbred racehorse cohorts, when categorised according to macroscopic OA severity, PCA identified that increased OA severity resulted in less variation between samples (Figures S11A and S12A). However, this was not evident when categorised according to microscopic OA grading. For mixed breeds native SF samples categorised according to macroscopic OA grading, the abundance of three uncharacterised proteins and immunoglobulin kappa constant was lower for higher OA severity grades (Figure 2). Abundances of an uncharacterised protein (a member of the superfamily containing a leucine‐rich repeat) and apolipoprotein A1 (ApoA1) were elevated for low‐grade OA pathology but then returned towards baseline for higher OA severity grades. Microscopic OA categorisation for mixed breeds native SF as well as macroscopic and microscopic OA categorisation of Thoroughbred racehorse native SF samples did not identify any differentially abundant proteins. The proteomes of Thoroughbred racehorse native SF samples, categorised according to synovitis grade, were not separated via PCA (Figure S12C). However, the abundance of afamin was identified as differentially abundant between low and high‐grade synovitis, lower for higher‐grade synovitis severity (Figure 3). ProteoMiner™ processing of both mixed breeds and Thoroughbred racehorse SF did not identify distinct proteome clusters (PCA) for any OA or synovitis categorisations (Figures S13 and S14). However, LC–MS/MS analysis of ProteoMiner™ processed Thoroughbred racehorse SF did identify lower gelsolin concentrations within higher OA grades (Figure 4A). ProteoMiner™ processed mixed breeds SF found that lipopolysaccharide binding protein (LBP) levels were reduced for higher OA severity grades and ApoA1 levels were elevated for OA grades 1 and 2 when compared with lower OA grades, which was consistent with the native SF findings.

### 7.5 Semi‐tryptic peptide profiles

No semi‐tryptic peptides arising from ECM proteins were identified that were more abundant at higher OA grades and thus, no potential neopeptide biomarkers were identified. Neither for the mixed breeds nor Thoroughbred racehorse cohorts were distinct profiles identified between severities of OA (Figure S15).

### 7.6 NMR metabolomics and LC–MS/MS proteomics integration

**FIGURE 5:**
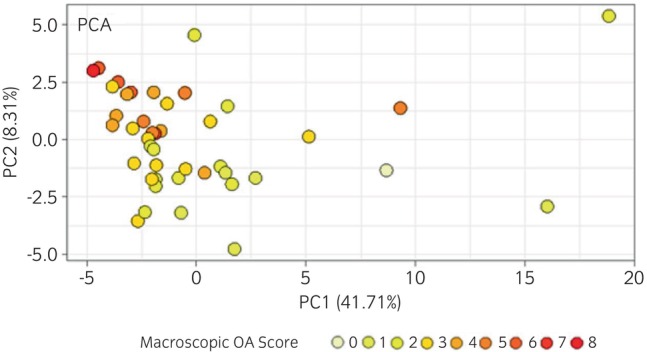
Principal component analysis (PCA) using 58 selected variables (proteins and metabolites) for the Thoroughbred racehorse equine synovial fluid combined metabolomic and proteomic datasets (*n* = 43) following batch correction, categorised according to macroscopic osteoarthritis (OA) score.

For the Thoroughbred racehorse dataset, correlation analysis of all variables (proteins and metabolites) identified 58 significant variables, with a range in correlation from −0.48 to 0.42 (Table S6). Using these selected variables, PCA identified less variation between samples with higher macroscopic OA scores, although their grouping could not clearly be distinguished from lower‐scoring samples (Figure 5). For the mixed breeds dataset, correlation analysis of all variables (proteins and metabolites) identified 32 significant variables, with a range in correlation from −0.44 to 0.49 (Table S7).

### 7.7 Uncharacterised proteins

BLAST analysis of amino acid sequences of 11 uncharacterised proteins included within this study identified various related characterised proteins. The characterised protein with the highest percentage amino acid sequence similarity for each uncharacterised protein is shown in Table S8.

## 8 DISCUSSION

Within this study, we have investigated equine SF from joints with varying OA severity using both NMR‐led metabolomics and MS‐based proteomics approaches. ^1^H NMR SF spectra were more variable between mixed breeds donors compared with the Thoroughbred racehorse donors. This is likely because the mixed breeds cohort is a more heterogeneous population, including age, breed, workloads and diet. The Thoroughbred racehorse is a much more controlled sample set, with all horses housed together, all the same breed, fed and trained similarly and all a similar age at euthanasia. These differentials may also reflect the different markers of interest identified between the mixed breeds and Thoroughbred racehorse cohorts. Proteomic analysis of mixed breeds samples identified several proteins that were able to discriminate between joints with OA grades of 0 and 1. Although these proteins were largely uncharacterised, BLAST analysis of the amino acid sequences of three uncharacterised proteins, decreasing in abundance with OA severity, identified high levels of similarity to immunoglobulin gamma 1 heavy chain constant region and immunoglobulin kappa chain V‐III region MOPC 63 proteins. However, abundances of these uncharacterised proteins could not be validated using antibody methodologies. Recently, a study investigating the SF proteome of a surgery‐induced OA model in rabbits identified reduced levels of immunoglobulin heavy chain protein compared with sham controls.^66^ Immunoglobulins have previously been identified within superficial articular cartilage layers of a proportion of OA patients, as well as elevated levels found within the synovial membrane of dogs diagnosed with cranial cruciate ligament rupture.^67^, ^68^ Thus, reduction of these immunoglobulins within the SF may be reflective of their translocation to surrounding articular tissues.

Glutamate is an excitatory amino acid neurotransmitter within the central nervous system, although evidence also suggests glutamate operates through intercellular signalling cascades, as an autocrine/paracrine factor, in non‐neuronal tissues.^69^ A self‐sufficient glutamate signalling machinery has been identified within chondrocytes, with a peripheral NMDA receptor proposed to have a role within inflammation and cartilage degradation.^70^ Within our study, the mixed breeds cohort showed a lower glutamate concentration in synovial fluid at higher OA severities (macroscopic OA grades 2 and 3 compared with grade 1). Previously, elevated levels of glutamate have been identified within OA SF of humans and within an OA rat models.^71^, ^72^, ^73^ However, no increase in glutamate was identified with equine OA SF using ^1^H NMR, although it is not clear whether glutamate was identified during this study.^23^ Therefore, at this stage, the relationship between synovial glutamate abundance and equine OA remains inconclusive.

Within this study, alanine levels were identified to be elevated in SF sampled from joints with higher grades of OA, which was also observed by Lacitignolia et al^23^ Previously, depleted alanine abundance has been identified within human OA cartilage using high‐resolution magic angle spinning NMR spectroscopy.^74^ As alanine is one of the main amino acid residues that constitute collagen, it may be that the reduction in alanine abundance identified within OA cartilage is resultant from the degradation of the cartilage collagen framework, which are subsequently released into the SF, resulting in the elevated synovial abundance within this study.^74^, ^75^

Gelsolin is a multifunctional, calcium ion‐regulated actin filament severing, capping, and nucleating protein involved in the determination of cell shape, secretion and chemotaxis.^76^, ^77^ Previous studies of gelsolin on different biological systems have identified gelsolin as a potential predictor of both inflammation and tissue injury.^78^, ^79^, ^80^ Within rheumatoid arthritis patients, reduced circulating levels of plasma gelsolin have been identified.^81^ Within this study, lower SF gelsolin concentrations were identified in joints with higher OA severity grades. These results are supported by a mouse model whereby gelsolin knockout mice resulted in arthritis exacerbation.^82^ Additionally, within a mouse model of pain and acute inflammation, exogenous delivery of gelsolin was identified to have effective analgesic and anti‐inflammatory properties.^83^ Exogenous gelsolin administration has also been shown to have chondroprotective properties, nullifying the effect of interleukin‐1β and OA SF on anabolic gene expression and increased glycosaminoglycan deposition in chondrocytes and protection of the integrity of murine cartilage following intra‐articular injection.^77^ However, within a cohort of horses with a clinical diagnosis of OA, lower SF concentrations of gelsolin have been reported.^84^ Therefore, further studies are required to further investigate the relationship between SF gelsolin concentration and clinical OA severity.

LBP is an endogenous protein which binds to lipopolysaccharides and catalytically delivers monomeric liposaccharides to the cluster of differentiation 14 (CD14) protein.^85^, ^86^ Previously, serum and synovial levels of LBP were not identified to be differentially expressed between human degenerative arthropathy patients and control samples.^87^ However, increased plasma LBP levels have recently been described to predict knee OA progression.^88^ Within this current study, the trends identified suggest a decreasing SF abundance of LBP with increasing OA severity. It has previously been identified that mononuclear cell activation, induced by lipopolysaccharides, is enhanced by low LBP concentrations.^89^ Activation of monocytes and macrophages by lipopolysaccharides leads to the secretion of tumour necrosis factor alpha and interleukin‐1 beta, two pro‐inflammatory cytokines which are central to OA pathogenesis.^90^, ^91^ Thus, decreasing synovial LBP levels may have a role in OA development.

Elevated SF concentrations of ApoA1 were identified for macroscopic OA grades 1 and 2 within the mixed breeds cohort, although abundance was lower for grade 3. Elevations in OA SF have previously been identified in horses and dogs.^32^, ^92^ ApoA1 is the primary protein component of high‐density lipoproteins (HDLs) and is involved in HDL binding to ATP‐binding cassette (ABC) transporters as well as being a lecithin cholesterol acyl transferase cofactor.^93^, ^94^, ^95^, ^96^ ApoA1 has previously been found to induce the expression of interleukin‐6, MMP‐1 and MMP‐3 in chondrocytes and synoviocytes through toll‐like receptor 4, with the same study identifying a dissociation between the relationship of ApoA1 and HDLs in OA SF.^97^

Synovitis has previously been identified as an important aspect of OA pathogenesis.^98^, ^99^, ^100^ This study identified a lower SF concentration of afamin in high‐grade synovitis compared with low‐grade. Similarly, reduced afamin levels have been recorded in equine OA SF compared with healthy joints.^32^ In a previous study, however, elevated levels of afamin were identified within human knee OA SF.^101^ Afamin is a vitamin E binding glycoprotein and a member of the albumin gene family.^102^ Afamin forms a 1:1 complex with various hydrophobic Wnt proteins, solubilising the proteins and producing a biologically active complex.^103^ A growing body of evidence has identified that the Wnt/β‐catenin signalling cascade is likely to have a central role within OA pathogenesis.^104^ Thus, a reduction in synovial abundance of afamin may be reflective of a translocation following Wnt solubilisation to surrounding articular tissues, that is, the synovium. However, it should be noted that a differential abundance of afamin was not identified when categorised according to OA severity.

### 8.1 Limitations

While this is the largest study of its type within the field, given the complexity of this study and the ‘omics’ analyses applied, the authors accept this will have resulted in an overall underpowering. Although semi‐quantitative scoring systems were used during this study, currently no generally accepted universal case definition or stratification methods have been validated for equine OA, and it is unclear how our stratification grades correlate with clinical OA severity, particularly for samples obtained from the abattoir. Due to a limited number of donors available for this study, we have included samples obtained from MTP joints in which MCP joints have also been sampled. Within this study, we have not performed a validation step on our PCA analyses to assess performance or stability. We would therefore recommend that further work building on the results of this study incorporates this approach. While not the purpose of our study, we are unable to statistically compare the Thoroughbred racehorse cohort to the mixed breeds cohort as OA severity has been scored using different scoring systems, due to the Thoroughbred racehorse cohort having been scored historically and the mixed breeds cohort being overall considerably older, which could therefore confound results. Samples from the two cohorts were also collected over different periods of time, spanning different years. Given the limited information available for most of these horses, we are also unable to determine whether certain horses within the mixed breeds population also had post‐traumatic OA or whether a proportion of the Thoroughbred racehorse population also had primary OA. Given that for a large proportion of joint samples the horse's sex is unknown, we are also unable to assess the impact that sex may have had on the results. In addition, for the mixed breeds cohort, we were unable to age‐match the samples across the OA severity stratification grades and therefore, further work is required to ensure these markers are true markers of OA severity opposed to age‐related markers.

### Conclusions

In conclusion, by stratifying equine OA severity through histological and macroscopic grading, we have identified a panel of SF metabolite and protein markers of interest which may be applicable to grading OA severity. Further research is required to correlate these markers to clinical OA severity and further explore their ability to aid an earlier diagnosis and support disease progression monitoring.

## FUNDING INFORMATION

Dr. James Anderson was funded through a Horse Trust PhD studentship (G1015) and Professor Mandy Peffers was funded through a Wellcome Trust Intermediate Clinical Fellowship (107 471/Z/15/Z). Software licences for data analysis used in the Shared Research Facility for NMR metabolomics were funded by the Medical Research Council (MRC) Clinical Research Capabilities and Technologies Initiative (MR/M009114/1). This work was also supported by the MRC and Versus Arthritis as part of the Medical Research Council Versus Arthritis Centre for Integrated Research into Musculoskeletal Ageing (CIMA) [MR/R502182/1] and a Technology Directorate Voucher (Faculty of Health and Life Sciences, University of Liverpool). The MRC Versus Arthritis Centre for Integrated Research into Musculoskeletal Ageing is a collaboration between the Universities of Liverpool, Sheffield and Newcastle.

## CONFLICT OF INTEREST STATEMENT

The authors declare no conflicts of interest.

## AUTHOR CONTRIBUTIONS

**James R. Anderson:** Investigation; writing – original draft; methodology; writing – review and editing; data curation; formal analysis. **Marie M. Phelan:** Writing – original draft; methodology; writing – review and editing; formal analysis; supervision. **Eva Caamaño‐Gutiérrez:** Writing – review and editing; methodology; formal analysis; data curation; writing – original draft. **Peter D. Clegg:** Conceptualization; funding acquisition; methodology; writing – review and editing; visualization; supervision. **Luis M. Rubio‐Martinez:** Writing – review and editing; supervision; resources. **Mandy J. Peffers:** Conceptualization; investigation; funding acquisition; writing – review and editing; methodology; formal analysis; project administration; supervision; resources; software; validation.

## DATA INTEGRITY STATEMENT

Dr. James Anderson has full access to all the data in the study and takes responsibility for the integrity of the data and the accuracy of the data analysis.

## ETHICAL ANIMAL RESEARCH

Collection of post‐mortem equine synovial fluid was approved by The University of Liverpool Veterinary Research Ethics Committee, approval reference VREC561. Research ethics committee oversight is not required by this journal for abattoir samples.

## INFORMED CONSENT

Hong Kong Jockey Club samples were collected under the regulations of the Hong Kong Jockey Club with owner consent. Samples collected from horses euthanised at The Philip Leverhulme Equine Hospital, University of Liverpool, were also collected with owner consent.

## PEER REVIEW

The peer review history for this article is available at https://www.webofscience.com/api/gateway/wos/peer-review/10.1111/evj.14490.

## Supporting information


**Data S1.** Methods S1. Liquid chromatography tandem mass spectrometry—Detailed methods.

**Figure S1.** Protocol for synovial fluid (SF) collection and processing prior to NMR metabolomic and LC–MS/MS proteomic analysis.

**Figure S2.** Parasagittal articular cartilage/subchondral bone wedge sections. (A) Blue box indicates the sampling site for metacarpal III on the medial condyle. (B) Blue box indicates the sampling site for metatarsal III on the lateral condyle. (C) Parasagittal articular cartilage/subchondral bone wedge sample dimensions.

**Figure S3.** Representative ion chromatograms for native synovial fluid following 16 h + 2 h trypsin digestion using a 90 min liquid chromatography (LC) gradient, ProteoMiner™ processed synovial fluid following a 4 h Lys‐C + 4 h trypsin digestion using a 60 min LC gradient and ProteoMiner™ processed synovial fluid following a 4 h Lys‐C + 16 h + 2 h trypsin digestion using a 120 min LC gradient.

**Figure S4.** Principal component analyses of mixed breeds and Thoroughbred (TB) equine synovial fluid NMR metabolomes and LC–MS/MS proteomes before and after application of a COMBAT batch correction. Mixed Breeds; metabolomics, *n* = 76, proteomics, *n* = 70; TB Racehorses; metabolomics, *n* = 56, proteomics, *n* = 53.

**Figure S5.** Examples of macroscopic and microscopic osteoarthritis‐related pathology scoring for the mixed breeds cohort using the equine OARSI scoring scale.^48^ Macroscopic scoring was conducted on the distal metacarpal III articular surface. (A) Grade 0, normal; (B) Grade 1, score 1 erosion; (C) Grade 2, score 1 erosion, score 1 wear line; (D) Grade 3, score 1 erosion, score 2 wear lines, score 2 palmar arthrosis. Microscopic scoring was conducted on parasagittal wedge sections of articular cartilage/subchondral bone stained with haematoxylin and eosin (H & E) or Safranin O (Saf O). (E) score 0 fissuring, score 0 focal cell loss, score 0 chondrone formation; (F) score 0 fissuring, score 1 focal cell loss, score 2 chondrone formations; (G) score 4 fissuring, score 4 Saf O uptake.

**Figure S6.** Examples of macroscopic and microscopic osteoarthritis pathology scoring for the Thoroughbred racehorse cohort using osteoarthritis‐related and palmar/plantar osteochondral disease (POD) pathology using published scoring scales.^18^, ^49^ Macroscopic scoring was conducted on the distal metacarpal III and metatarsal III articular surfaces. (A) Grade 0, normal; (B) Grade 1, score 1 POD, score 1 wear lines, score 1 linear fissures; (C) Grade 2, score 3 POD, score 2 wear lines, score 3 cartilage loss. Microscopic scoring was conducted on parasagittal wedge sections of articular cartilage/subchondral bone stained with haematoxylin and eosin (H & E). (E) score 3 structure, score 0 chondrocyte density, score 2 cell cloning; (F) score 0 structure, score 2 chondrocyte density, score 2 cell cloning; (G) score 4 structure, score 1 chondrocyte density, score 0 cell cloning.

**Figure S7.** Correlation between microscopic and macroscopic osteoarthritis (OA) scores for mixed breeds (*n* = 41) and Thoroughbred (TB) racehorses (*n* = 41) cohorts using a Pearson correlation coefficient.

**Figure S8.** Quantile plots of (A) mixed breeds (*n* = 83) and (B) Thoroughbred (TB) racehorses (*n* = 52) synovial fluid (SF) NMR spectra. The median spectra are depicted by a black line, with variation from the median spectral plot depicted by a yellow to red scale. The full spectral range is shown (8.56–0 ppm) with a more detailed region inset (4–3.1 ppm). Spectral regions 3.681–3.643 ppm and 1.201–1.162 ppm have been removed due to ethanol contamination.

**Figure S9.** Principal component analysis (PCA) of metabolite profiles of mixed‐breed equine synovial fluid categorised according to (A) OARSI microscopic (*n* = 70) and (B) OARSI macroscopic (*n* = 76) grading.

**Figure S10.** Principal component analysis (PCA) of metabolite profiles of Thoroughbred (TB) racehorse synovial fluid categorised according to (A) OARSI microscopic (*n* = 50), (B) OARSI macroscopic (n = 50) and (C) synovitis (*n* = 56) grading.

**Figure S11.** Principal component analysis (PCA) of the mixed breeds native equine synovial fluid proteome categorised by (A) macroscopic osteoarthritis (OA) grade and (B) microscopic OA grade using LC–MS/MS. Macroscopic OA, *n* = 66; microscopic OA, *n* = 70.

**Figure S12.** Principal component analysis (PCA) of the Thoroughbred (TB) racehorse native equine synovial fluid proteome profile categorised by (A) macroscopic osteoarthritis (OA) grade (*n* = 49), (B) microscopic OA grade (*n* = 49) and (C) synovitis grade (*n* = 53) using LC–MS/MS.

**Figure S13.** Principal component analysis of mixed breeds ProteoMiner™ processed (16 h + 2 h trypsin digestion) synovial fluid proteome categorised by (A) macroscopic OA grade (*n* = 60) and (B) microscopic OA grade (*n* = 64) using LC–MS/MS.

**Figure S14.** Principal component analysis of the Thoroughbred (TB) racehorse ProteoMiner™ processed (16 h + 2 h trypsin digestion) synovial fluid proteome categorised by (A) macroscopic OA grade (*n* = 47), (B) microscopic OA grade (*n* = 45) and (C) synovitis grade (*n* = 53) using LC–MS/MS.

**Figure S15.** Principal component analysis (PCA) of equine synovial fluid semi‐tryptic peptide profiles categorised according to (A) microscopic osteoarthritis (*n* = 62) and (B) macroscopic osteoarthritis (*n* = 59) grading for the mixed breeds sample set and (C) microscopic osteoarthritis (*n* = 46), (D) macroscopic osteoarthritis (*n* = 46) and (E) synovitis (*n* = 53) grading for the Thoroughbred (TB) racehorse sample set.

**Table S1.** Macroscopic osteoarthritis scoring of distal metacarpal III for the mixed breeds sample set.

**Table S2.** Microscopic osteoarthritis scoring of distal metacarpal III for the mixed breeds sample set.

**Table S3.** Macroscopic osteoarthritis scoring of distal metacarpal III or metatarsal III for the Thoroughbred racehorse sample set.

**Table S4.** Microscopic osteoarthritis scoring of distal metacarpal III or metatarsal III for the Thoroughbred racehorse sample set.

**Table S5.** Synovitis scoring of distal metacarpal III or metatarsal III for the Thoroughbred racehorse sample set.

**Table S6.** Correlation of each variable (proteins and metabolites) to macroscopic OA score for the Thoroughbred racehorse synovial fluid integrated dataset. *p* < 0.05.

**Table S7.** Correlation of each variable (proteins and metabolites) to macroscopic OA score for the mixed‐breed synovial fluid integrated dataset. *p* < 0.05.

**Table S8.** BLAST analysis of amino acid sequences of uncharacterised proteins included within this study, identifying the characterised protein with the highest percentage amino acid sequence similarity for each uncharacterised protein.

## Data Availability

The proteomics data that support the findings of this study are openly available in the ProteomeXchange Consortium via the PRIDE partner repository with the dataset identifier PXD019842. The metabolomics data that support the findings of this study are openly available in the MetaboLights repository (https://www.ebi.ac.uk/metabolights/), reference number MTBLS1645.
