# Supplementary material for: Metabolomic and proteomic stratification of equine osteoarthritis
Source: Equine Vet J. 2025 Feb 19;57(5):1204–18. doi: 10.1111/evj.14490 (PMC12326899; doi:10.1111/evj.14490)
Supplement: Supplementary file 1 — Data S1. Methods S1. Liquid chromatography tandem mass spectrometry—Detailed methods. [file EVJ-57-1204-s024.pdf]

Equine Veterinary Journal BEVA

Methods S1:

Liquid Chromatography Tandem Mass Spectrometry - Detailed Methods

Tryptic digests were diluted 5-fold in 0.1% (v/v) trifluoroacetic acid (TFA) and 3% (v/v) acetonitrile and analysed individually, in a random order, via liquid chromatography tandem mass spectrometry (LC-MS/MS) using a 60, 90 or 120 min liquid chromatography (LC) gradient as stated. A Q Exactive^{TM} HF quadrupole-Orbitrap mass spectrometer (Thermo Scientific) coupled to a Dionex Ultimate 3000 RSLC nano-liquid chromatograph (Thermo Scientific) was used for data-dependent LC-MS/MS analyses. Digests were loaded onto a trapping column (Acclaim PepMap 100, C18, 20 mm x 75 μm) using a loading buffer of 0.1% (v/v) TFA and 2% (v/v) acetonitrile in water for 3 min at a flow rate of 5 μl min^{-1}. The trapping column was then set in-line with an analytical column (Easy-Spray PepMap® C18, 15 cm x 75 μm, 2 μm) with peptide elution carried out using a linear gradient of 96.2% A (0.1% (v/v) formic acid):3.8% B (0.1 % (v/v) formic acid in water:acetonitrile (80:20) (v/v)) to 50% A:50% B over 30, 60 0r 90 min at a flow rate of 300 nl min^{-1}, followed by washing at 1 % A:99% B for 5 min and re-equilibration of the column to starting conditions. The Q Exactive^{TM} was operated in data dependent positive (ESI+) mode with survey scans between *m/z* 300-2000 acquired at a mass resolution of 70,000 (full width at half maximum) at *m/z* 200 after accumulation of ions to $1x10^6$ target value based on predictive automatic gain control values from the previous full scan. The 10 most intense precursor ions with charge states of between 2+ and 5+ were selected for MS/MS with an isolation window of 2 *m/z* units. Higher-energy collisional dissociation (HCD) was used to fragment peptides using normalised collision energy of 30% with a maximum injection time of 100 ms. Dynamic exclusion of *m/z* values to prevent repeated fragmentation of the same peptide was used with an exclusion time of 20 s.
