# Supplementary material for: Metabolomic and proteomic stratification of equine osteoarthritis
Source: Equine Vet J. 2025 Feb 19;57(5):1204–18. doi: 10.1111/evj.14490 (PMC12326899; doi:10.1111/evj.14490)

Equine Veterinary Journal BEVA

**Figure S1.** Protocol for synovial fluid (SF) collection and processing prior to NMR metabolomic and LC-MS/MS proteomic analysis. MCP = metacarpophalangeal, MTP = metatarsophalangeal.

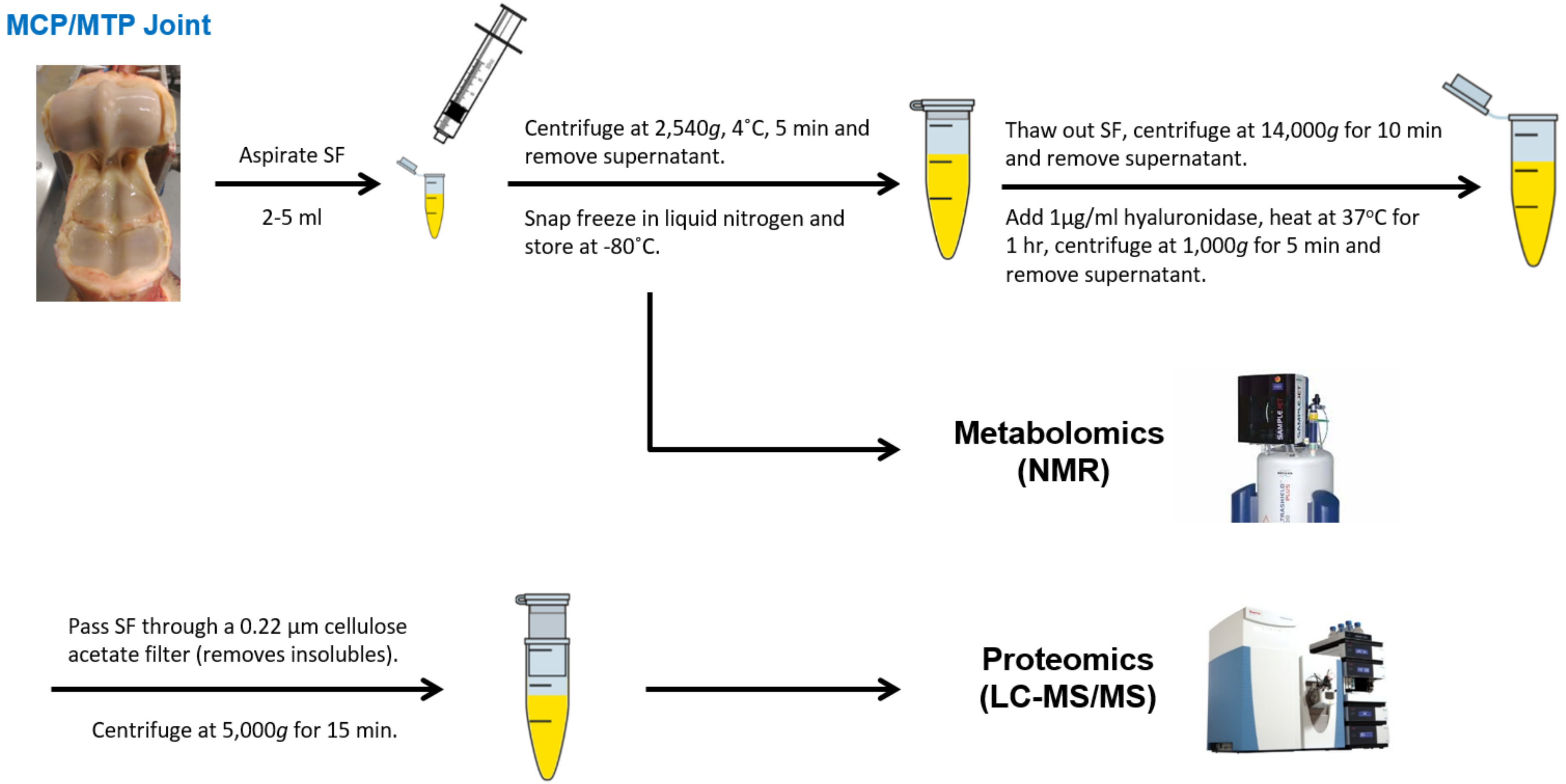

Supplement: Supplementary file 2 — Figure S1. Protocol for synovial fluid (SF) collection and processing prior to NMR metabolomic and LC–MS/MS proteomic analysis. [file EVJ-57-1204-s005.pdf]
