# Supplementary material for: Metabolomic and proteomic stratification of equine osteoarthritis
Source: Equine Vet J. 2025 Feb 19;57(5):1204–18. doi: 10.1111/evj.14490 (PMC12326899; doi:10.1111/evj.14490)

Equine Veterinary Journal
BEVA

Figure S2. Parasagittal articular cartilage/subchondral bone wedge sections. (A) Blue box indicates sampling site for metacarpal III on the medial condyle. (B) Blue box indicates sampling site for metatarsal III on the lateral condyle. (C) Parasagittal articular cartilage/subchondral bone wedge sample dimensions.

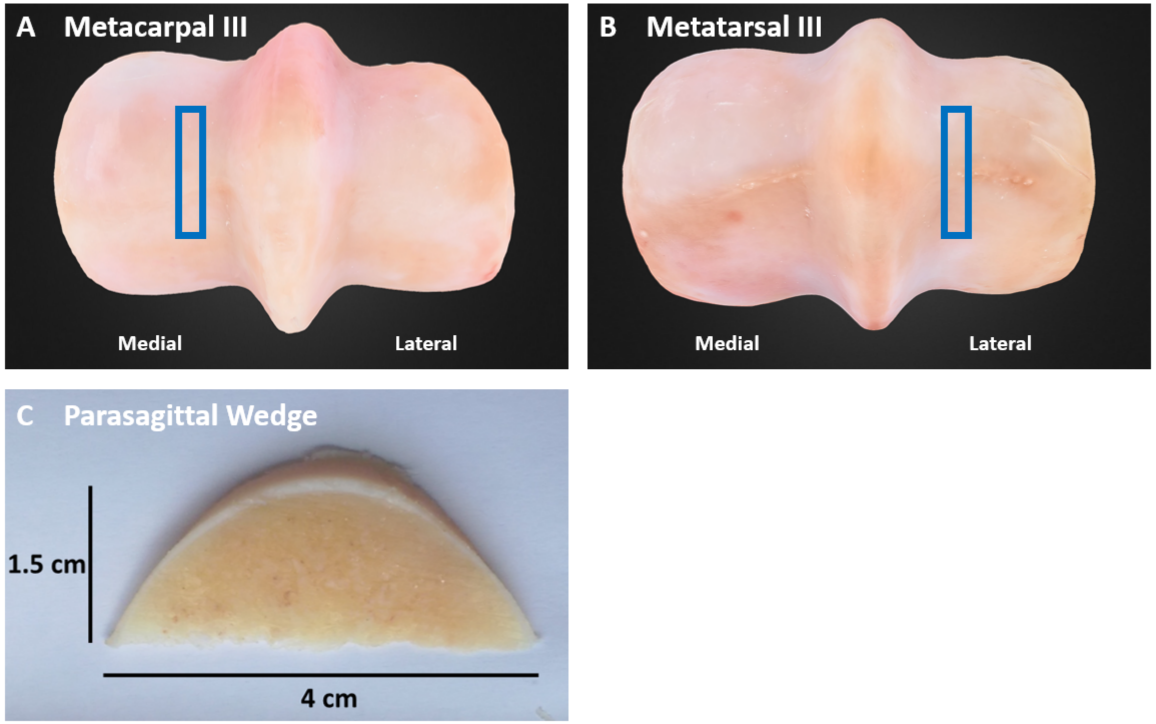

Supplement: Supplementary file 3 — Figure S2. Parasagittal articular cartilage/subchondral bone wedge sections. (A) Blue box indicates the sampling site for metacarpal III on the medial condyle. (B) Blue box indicates the sampling site for metatarsal III on the lateral condyle. (C) Parasagittal articular cartilage/subchondral bone wedge sample dimensions. [file EVJ-57-1204-s012.pdf]
