# Supplementary material for: Metabolomic and proteomic stratification of equine osteoarthritis
Source: Equine Vet J. 2025 Feb 19;57(5):1204–18. doi: 10.1111/evj.14490 (PMC12326899; doi:10.1111/evj.14490)

Equine Veterinary Journal BEVA

Figure S3. Representative ion chromatograms for (A) native synovial fluid (SF) following 16hr + 2hr trypsin digestion using a 90 min liquid chromatography (LC) gradient, (B) ProteoMiner^{TM} processed SF following a 4hr Lys-C + 4hr trypsin digestion using a 60 min LC gradient and (C) ProteoMiner^{TM} processed SF following a 4hr Lys-C + 16hr + 2hr trypsin digestion using a 120 min LC gradient.

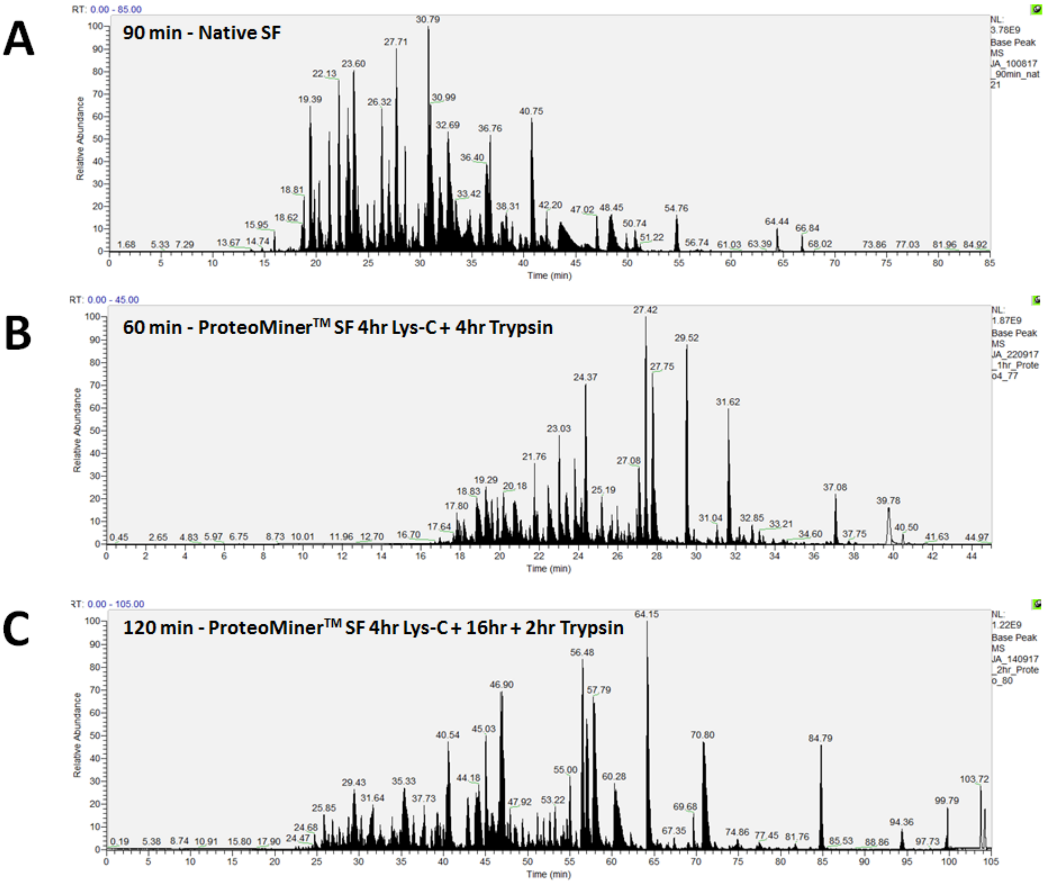

Supplement: Supplementary file 4 — Figure S3. Representative ion chromatograms for native synovial fluid following 16 h + 2 h trypsin digestion using a 90 min liquid chromatography (LC) gradient, ProteoMiner™ processed synovial fluid following a 4 h Lys‐C + 4 h trypsin digestion using a 60 min LC gradient and ProteoMiner™ processed synovial fluid following a 4 h Lys‐C + 16 h + 2 h trypsin digestion using a 120 min LC gradient. [file EVJ-57-1204-s020.pdf]
