# Supplementary material for: Metabolomic and proteomic stratification of equine osteoarthritis
Source: Equine Vet J. 2025 Feb 19;57(5):1204–18. doi: 10.1111/evj.14490 (PMC12326899; doi:10.1111/evj.14490)

Equine Veterinary Journal BEVA

Figure S4. Principal component analyses of mixed breeds and Thoroughbred (TB) equine synovial fluid NMR metabolomes and LC-MS/MS proteomes before and after application of a COMBAT batch correction. Mixed Breeds; metabolomics, n=76, proteomics, n=70, TB Racehorses; metabolomics, n=56, proteomics, n=53.

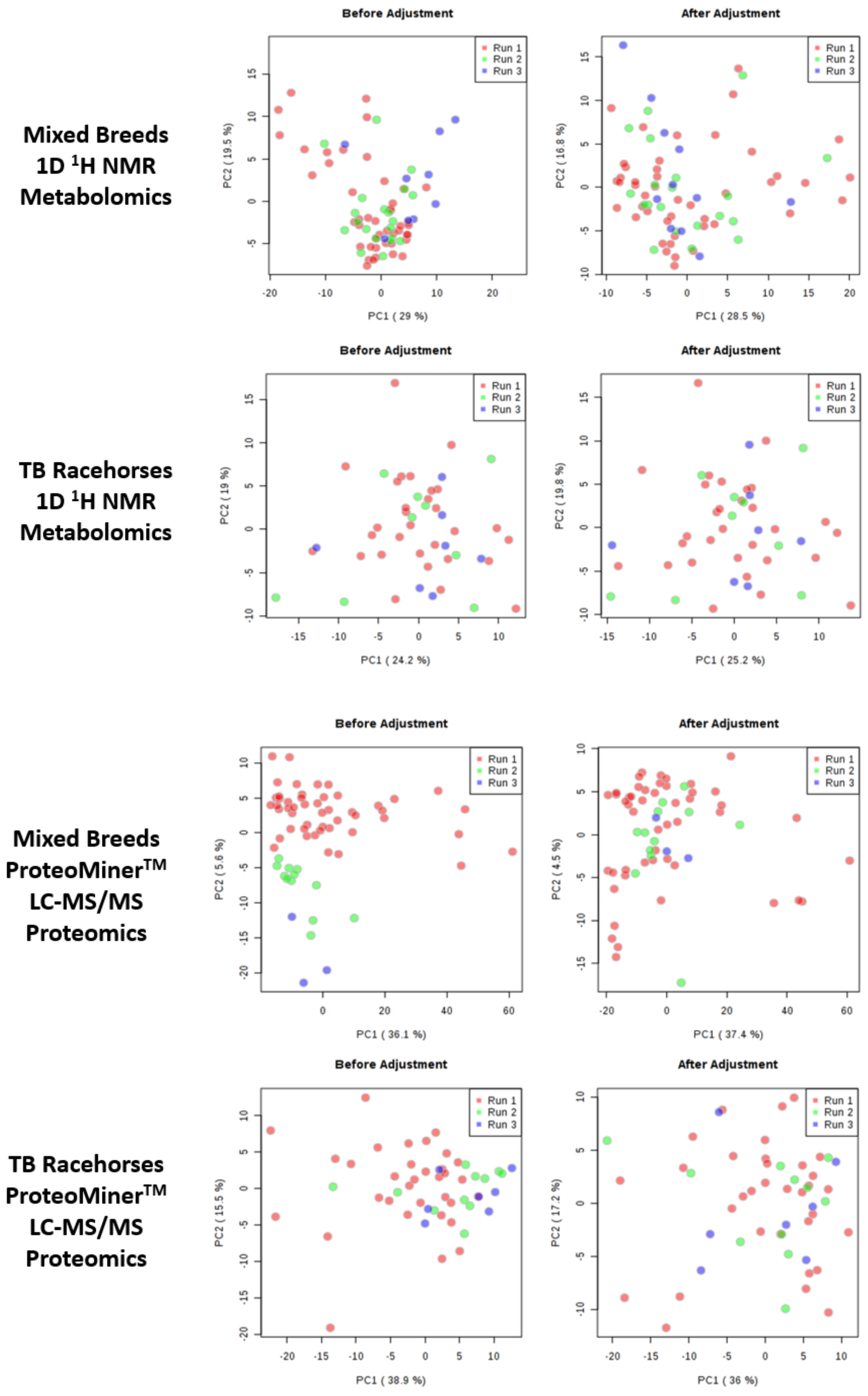

Supplement: Supplementary file 5 — Figure S4. Principal component analyses of mixed breeds and Thoroughbred (TB) equine synovial fluid NMR metabolomes and LC–MS/MS proteomes before and after application of a COMBAT batch correction. Mixed Breeds; metabolomics, n = 76, proteomics, n = 70; TB Racehorses; metabolomics, n = 56, proteomics, n = 53. [file EVJ-57-1204-s017.pdf]
