# Supplementary material for: Metabolomic and proteomic stratification of equine osteoarthritis
Source: Equine Vet J. 2025 Feb 19;57(5):1204–18. doi: 10.1111/evj.14490 (PMC12326899; doi:10.1111/evj.14490)

Equine Veterinary Journal
BEVA

Figure S5. Examples of macroscopic and microscopic osteoarthritis related pathology scoring for the mixed breeds cohort using the equine OARSI scoring scale (McIlwraith et al., 2010 [48]). Macroscopic scoring was conducted on the distal metacarpal III articular surface. (A) Grade 0, normal; (B) Grade 1, score 1 erosions; (C) Grade 2, score 1 erosions, score 1 wear lines; (D) Grade 3, score 1 erosions, score 2 wear lines, score 2 palmar arthrosis. Microscopic scoring was conducted on parasagittal wedge sections of articular cartilage/subchondral bone stained with haematoxylin and eosin (H & E) or Safranin O (Saf O). (E) score 0 fissuring, score 0 focal cell loss, score 0 chondrone formation; (F) score 0 fissuring, score 1 focal cell loss, score 2 chondrone formation; (G) score 4 fissuring, score 4 Saf O uptake.

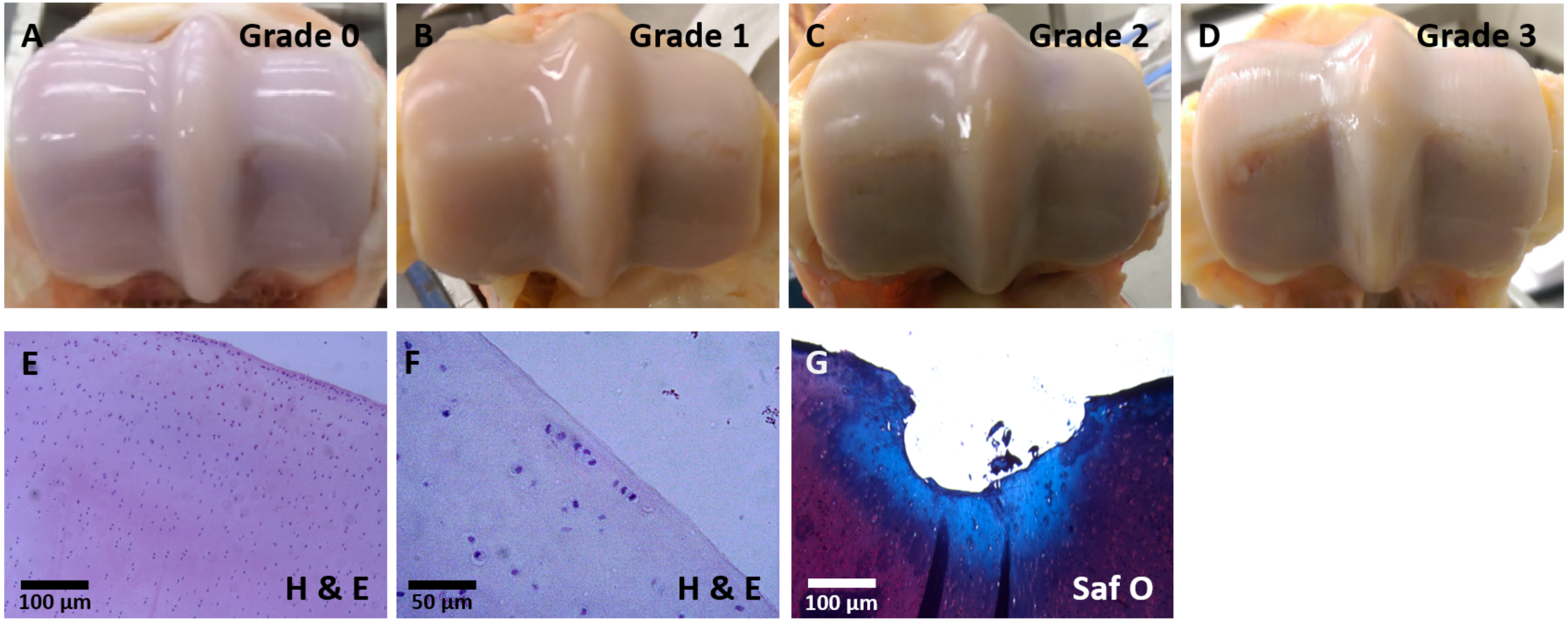

Supplement: Supplementary file 6 — Figure S5. Examples of macroscopic and microscopic osteoarthritis‐related pathology scoring for the mixed breeds cohort using the equine OARSI scoring scale. 48 Macroscopic scoring was conducted on the distal metacarpal III articular surface. (A) Grade 0, normal; (B) Grade 1, score 1 erosion; (C) Grade 2, score 1 erosion, score 1 wear line; (D) Grade 3, score 1 erosion, score 2 wear lines, score 2 palmar arthrosis. Microscopic scoring was conducted on parasagittal wedge sections of articular cartilage/subchondral bone stained with haematoxylin and eosin (H & E) or Safranin O (Saf O). (E) score 0 fissuring, score 0 focal cell loss, score 0 chondrone formation; (F) score 0 fissuring, score 1 focal cell loss, score 2 chondrone formations; (G) score 4 fissuring, score 4 Saf O uptake. [file EVJ-57-1204-s022.pdf]
