# Supplementary material for: Metabolomic and proteomic stratification of equine osteoarthritis
Source: Equine Vet J. 2025 Feb 19;57(5):1204–18. doi: 10.1111/evj.14490 (PMC12326899; doi:10.1111/evj.14490)

Equine Veterinary Journal BEVA

Figure S6. Examples of macroscopic and microscopic osteoarthritis pathology scoring for the Thoroughbred racehorse cohort using osteoarthritis related and palmar/plantar osteochondral disease (POD) pathology using published scoring scales (Barr et al., 2009 [18]; Little et al., 2010 [49]). Macroscopic scoring was conducted on the distal metacarpal III and metatarsal III articular surfaces. (A) Grade 0, normal; (B) Grade 1, score 1 POD, score 1 wear lines, score 1 linear fissures; (C) Grade 2, score 3 POD, score 2 wear lines, score 3 cartilage loss. Microscopic scoring was conducted on parasagittal wedge sections of articular cartilage/subchondral bone stained with haematoxylin and eosin (H & E). (E) score 3 structure, score 0 chondrocyte density, score 2 cell cloning; (F) score 0 structure, score 2 chondrocyte density, score 2 cell cloning; (G) score 4 structure, score 1 chondrocyte density, score 0 cell cloning.

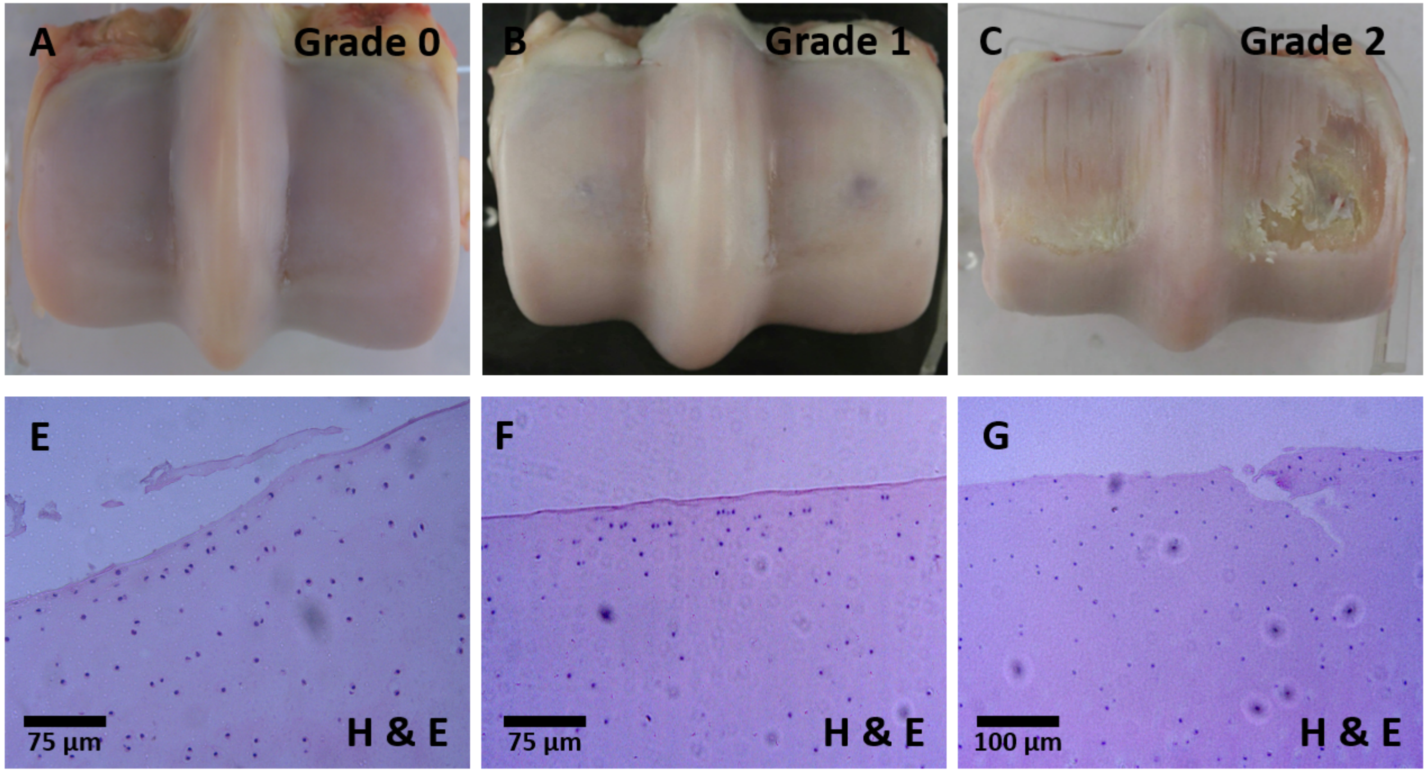

Supplement: Supplementary file 7 — Figure S6. Examples of macroscopic and microscopic osteoarthritis pathology scoring for the Thoroughbred racehorse cohort using osteoarthritis‐related and palmar/plantar osteochondral disease (POD) pathology using published scoring scales. 18 , 49 Macroscopic scoring was conducted on the distal metacarpal III and metatarsal III articular surfaces. (A) Grade 0, normal; (B) Grade 1, score 1 POD, score 1 wear lines, score 1 linear fissures; (C) Grade 2, score 3 POD, score 2 wear lines, score 3 cartilage loss. Microscopic scoring was conducted on parasagittal wedge sections of articular cartilage/subchondral bone stained with haematoxylin and eosin (H & E). (E) score 3 structure, score 0 chondrocyte density, score 2 cell cloning; (F) score 0 structure, score 2 chondrocyte density, score 2 cell cloning; (G) score 4 structure, score 1 chondrocyte density, score 0 cell cloning. [file EVJ-57-1204-s006.pdf]
