# Supplementary material for: Metabolomic and proteomic stratification of equine osteoarthritis
Source: Equine Vet J. 2025 Feb 19;57(5):1204–18. doi: 10.1111/evj.14490 (PMC12326899; doi:10.1111/evj.14490)

Equine Veterinary Journal BEVA

Figure S7. Correlation between microscopic and macroscopic osteoarthritis (OA) scores for mixed breeds (n=41) and Thoroughbred (TB) racehorse (n=41) cohorts using a Pearson correlation coefficient.

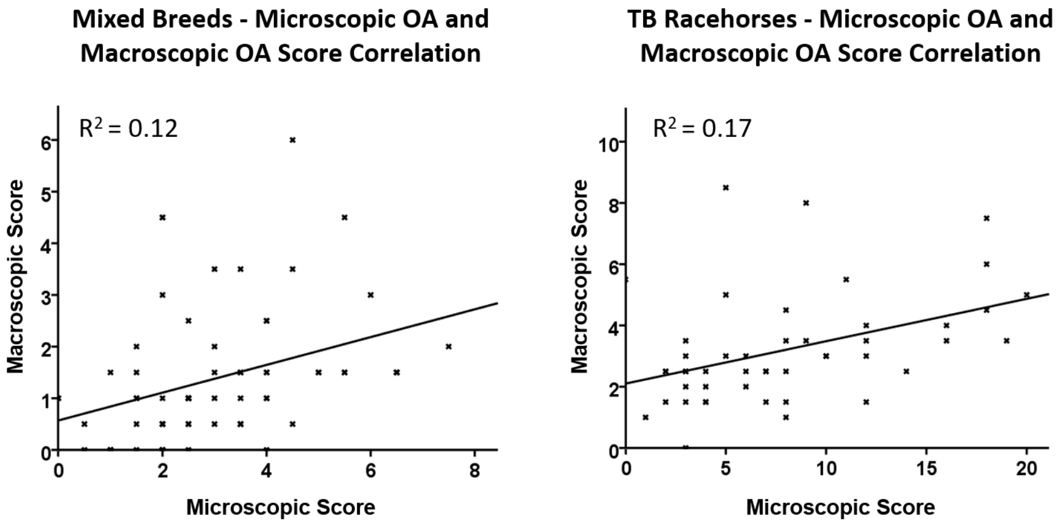

Supplement: Supplementary file 8 — Figure S7. Correlation between microscopic and macroscopic osteoarthritis (OA) scores for mixed breeds (n = 41) and Thoroughbred (TB) racehorses (n = 41) cohorts using a Pearson correlation coefficient. [file EVJ-57-1204-s016.pdf]
