# Supplementary material for: Metabolomic and proteomic stratification of equine osteoarthritis
Source: Equine Vet J. 2025 Feb 19;57(5):1204–18. doi: 10.1111/evj.14490 (PMC12326899; doi:10.1111/evj.14490)

Equine Veterinary Journal BEVA

Figure S8. Quantile plots of (A) mixed breeds (n=83) and (B) Thoroughbred (TB) racehorse (n=52) synovial fluid (SF) NMR spectra. The median spectra are depicted by a black line, with variation from the median spectral plot depicted by a yellow to red scale. The full spectral range is shown (8.56-0 ppm) with a more detailed region inset (4-3.1 ppm). Spectral regions 3.681-3.643 ppm and 1.201-1.162 ppm have been removed due to ethanol contamination.

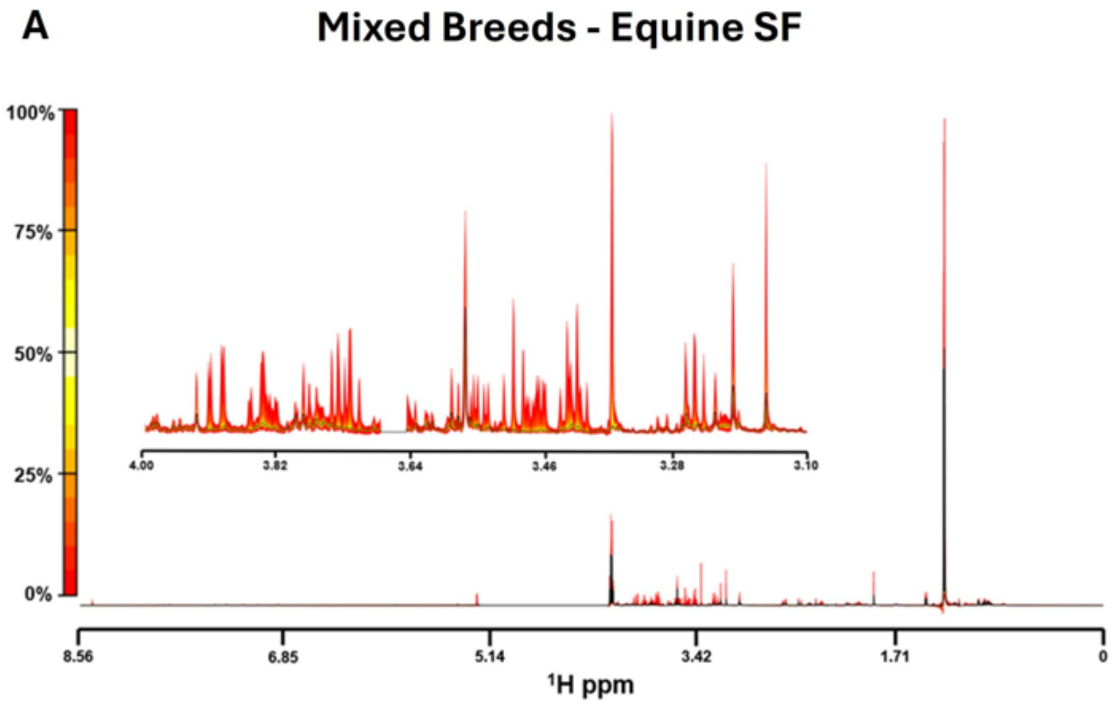

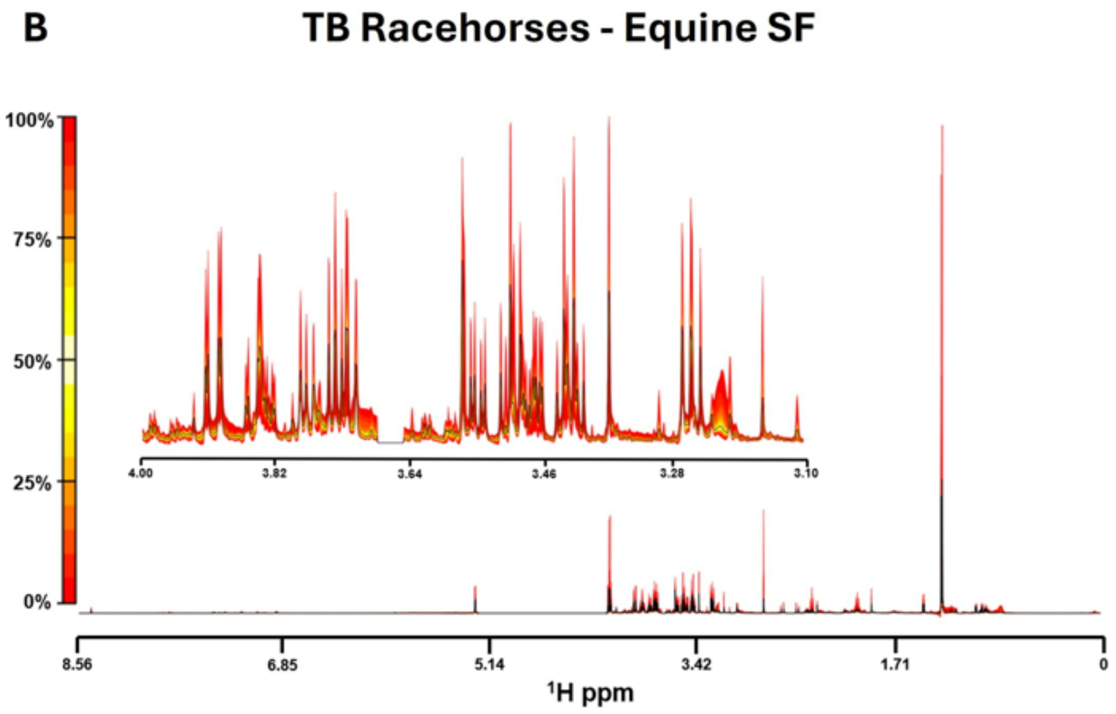

Supplement: Supplementary file 9 — Figure S8. Quantile plots of (A) mixed breeds (n = 83) and (B) Thoroughbred (TB) racehorses (n = 52) synovial fluid (SF) NMR spectra. The median spectra are depicted by a black line, with variation from the median spectral plot depicted by a yellow to red scale. The full spectral range is shown (8.56–0 ppm) with a more detailed region inset (4–3.1 ppm). Spectral regions 3.681–3.643 ppm and 1.201–1.162 ppm have been removed due to ethanol contamination. [file EVJ-57-1204-s014.pdf]
