# Supplementary material for: Metabolomic and proteomic stratification of equine osteoarthritis
Source: Equine Vet J. 2025 Feb 19;57(5):1204–18. doi: 10.1111/evj.14490 (PMC12326899; doi:10.1111/evj.14490)

Equine Veterinary Journal BEVA

Figure S9. Principal component analysis (PCA) of metabolite profiles of mixed breeds equine synovial fluid categorised according to (A) OARSI microscopic (n=70) and (B) OARSI macroscopic (n=76) grading.

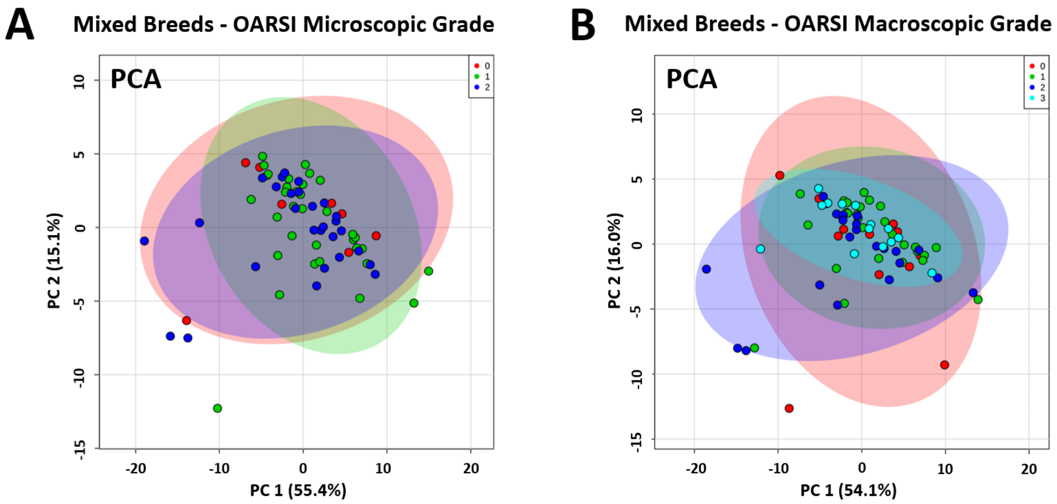

Supplement: Supplementary file 10 — Figure S9. Principal component analysis (PCA) of metabolite profiles of mixed‐breed equine synovial fluid categorised according to (A) OARSI microscopic (n = 70) and (B) OARSI macroscopic (n = 76) grading. [file EVJ-57-1204-s013.pdf]
