# Supplementary material for: Metabolomic and proteomic stratification of equine osteoarthritis
Source: Equine Vet J. 2025 Feb 19;57(5):1204–18. doi: 10.1111/evj.14490 (PMC12326899; doi:10.1111/evj.14490)

Equine Veterinary Journal
BEVA

Figure S10. Principal component analysis (PCA) of metabolite profiles of Thoroughbred (TB) racehorse synovial fluid categorised according to (A) OARSI microscopic (n=50), (B) OARSI macroscopic (n=50) and (C) synovitis (n=56) grading.

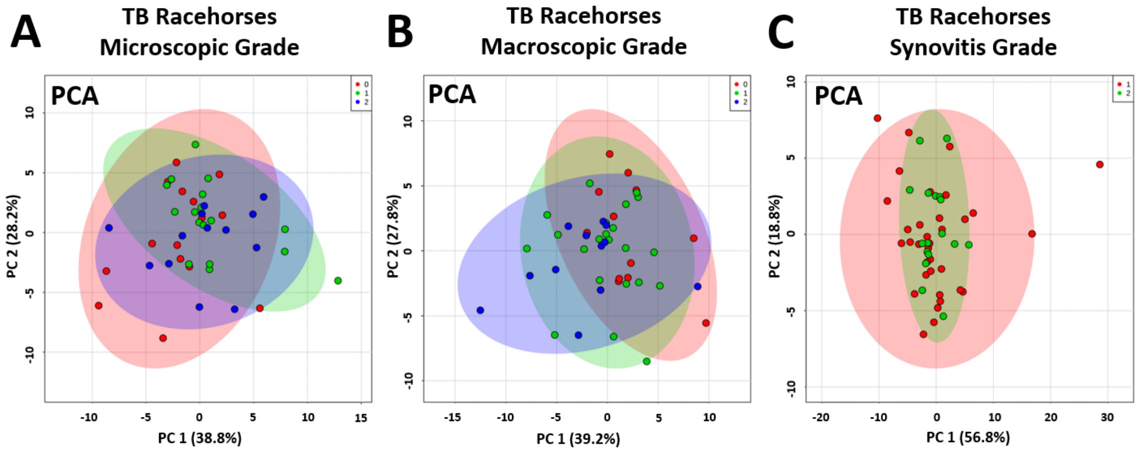

Supplement: Supplementary file 11 — Figure S10. Principal component analysis (PCA) of metabolite profiles of Thoroughbred (TB) racehorse synovial fluid categorised according to (A) OARSI microscopic (n = 50), (B) OARSI macroscopic (n = 50) and (C) synovitis (n = 56) grading. [file EVJ-57-1204-s001.pdf]
