# Supplementary material for: Metabolomic and proteomic stratification of equine osteoarthritis
Source: Equine Vet J. 2025 Feb 19;57(5):1204–18. doi: 10.1111/evj.14490 (PMC12326899; doi:10.1111/evj.14490)

Equine Veterinary Journal
BEVA

Figure S11. Principal component analysis (PCA) of the mixed breeds native equine synovial fluid proteome categorised by (A) macroscopic osteoarthritis (OA) grade and (B) microscopic OA grade using LC-MS/MS. Macroscopic OA, n=66, Microscopic OA, n=70.

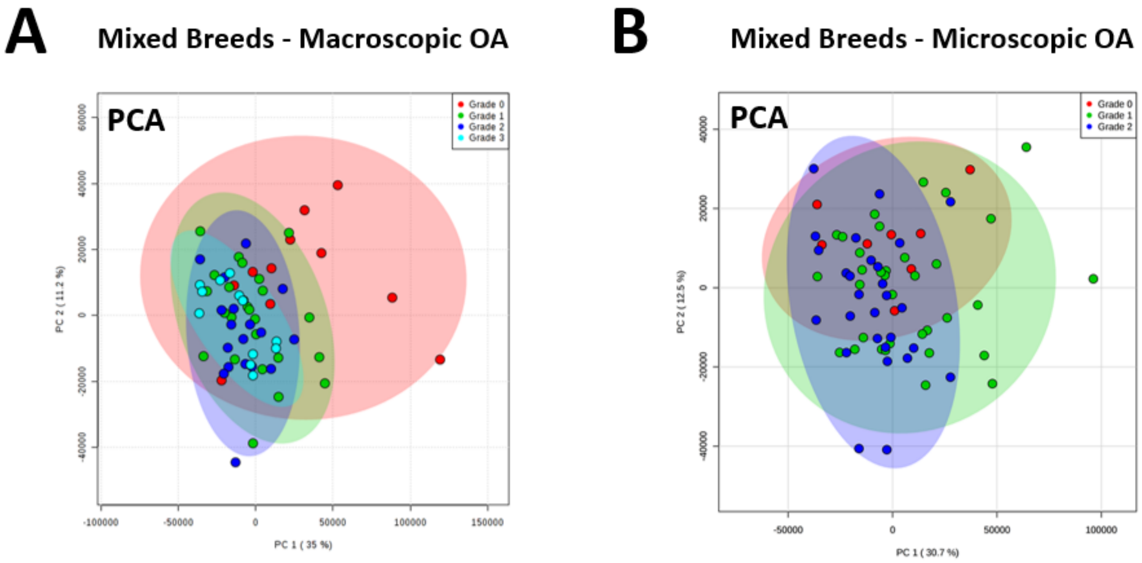

Supplement: Supplementary file 12 — Figure S11. Principal component analysis (PCA) of the mixed breeds native equine synovial fluid proteome categorised by (A) macroscopic osteoarthritis (OA) grade and (B) microscopic OA grade using LC–MS/MS. Macroscopic OA, n = 66; microscopic OA, n = 70. [file EVJ-57-1204-s015.pdf]
