# Supplementary material for: Metabolomic and proteomic stratification of equine osteoarthritis
Source: Equine Vet J. 2025 Feb 19;57(5):1204–18. doi: 10.1111/evj.14490 (PMC12326899; doi:10.1111/evj.14490)

Figure S12. Principal component analysis (PCA) of the Thoroughbred (TB) racehorse native equine synovial fluid proteome profile categorised by (A) macroscopic osteoarthritis (OA) grade (n=49), (B) microscopic OA grade (n=49) and (C) synovitis grade (n=53) using LC-MS/MS.

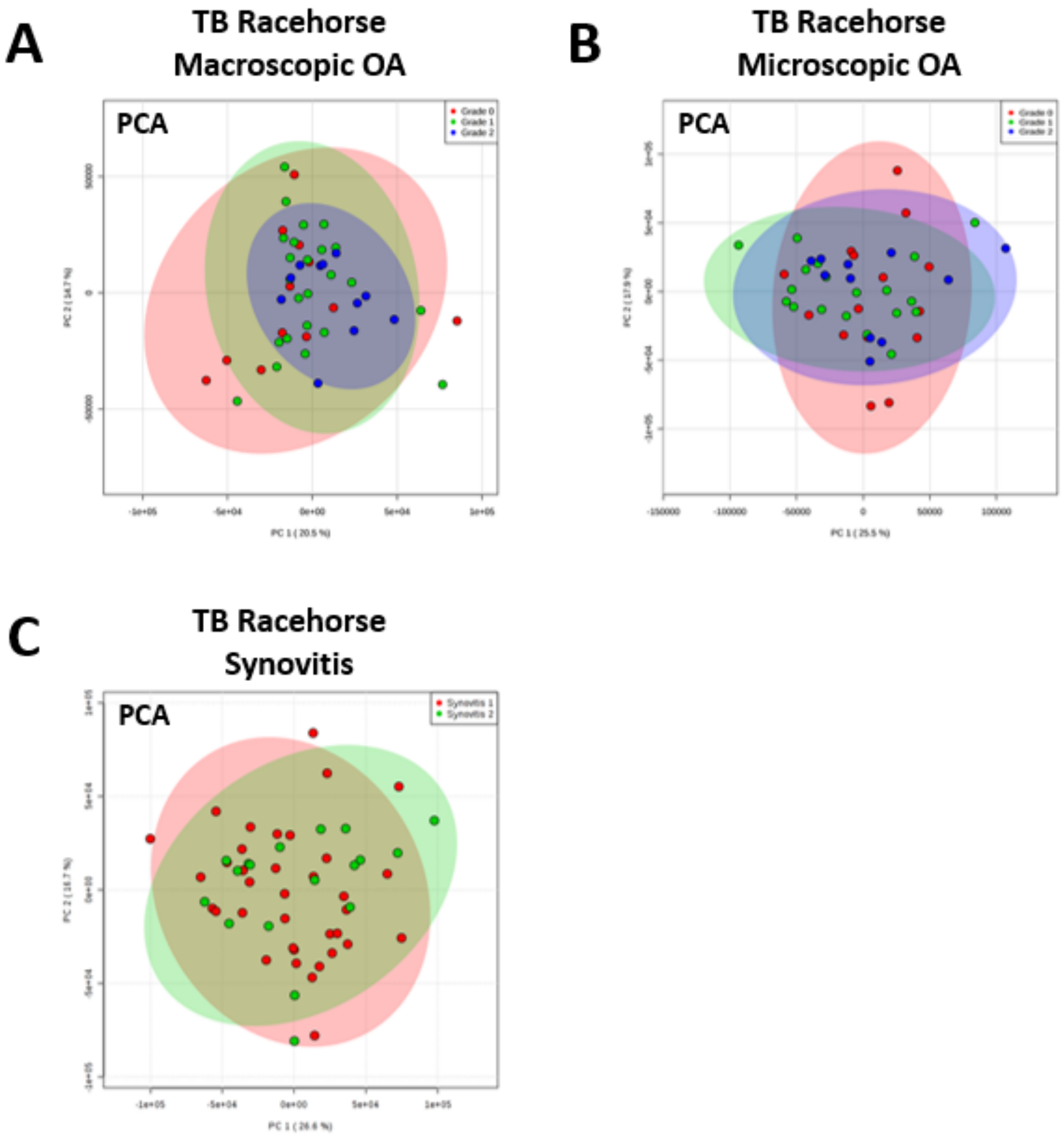

Supplement: Supplementary file 13 — Figure S12. Principal component analysis (PCA) of the Thoroughbred (TB) racehorse native equine synovial fluid proteome profile categorised by (A) macroscopic osteoarthritis (OA) grade (n = 49), (B) microscopic OA grade (n = 49) and (C) synovitis grade (n = 53) using LC–MS/MS. [file EVJ-57-1204-s018.pdf]
