# Supplementary material for: Metabolomic and proteomic stratification of equine osteoarthritis
Source: Equine Vet J. 2025 Feb 19;57(5):1204–18. doi: 10.1111/evj.14490 (PMC12326899; doi:10.1111/evj.14490)

Equine Veterinary Journal

BEVA

Figure S13. Principal component analysis of mixed breeds ProteoMiner^{TM} processed (16hr + 2hr trypsin digestion) synovial fluid proteome categorised by (A) macroscopic OA grade (n=60) and (B) microscopic OA grade (n=64) using LC-MS/MS.

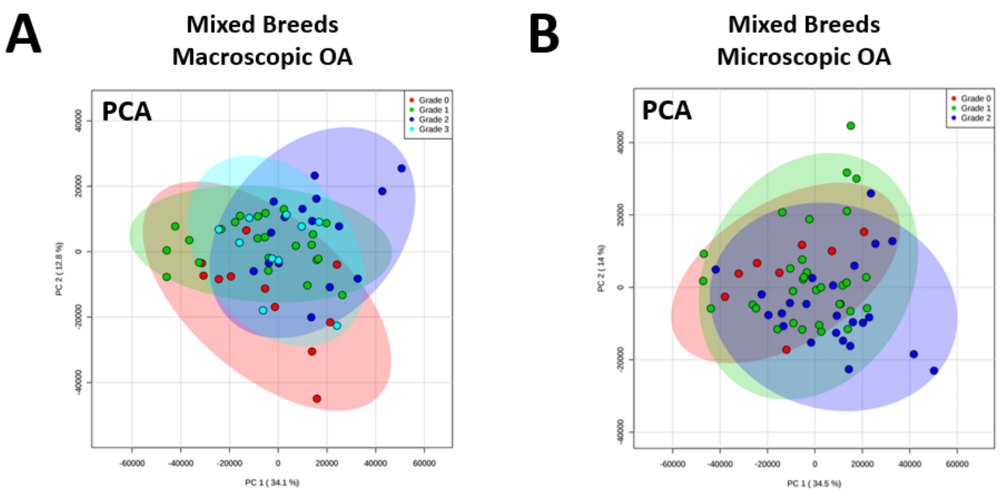

Supplement: Supplementary file 14 — Figure S13. Principal component analysis of mixed breeds ProteoMiner™ processed (16 h + 2 h trypsin digestion) synovial fluid proteome categorised by (A) macroscopic OA grade (n = 60) and (B) microscopic OA grade (n = 64) using LC–MS/MS. [file EVJ-57-1204-s004.pdf]
