# Supplementary material for: Metabolomic and proteomic stratification of equine osteoarthritis
Source: Equine Vet J. 2025 Feb 19;57(5):1204–18. doi: 10.1111/evj.14490 (PMC12326899; doi:10.1111/evj.14490)

Equine Veterinary Journal BEVA

Figure S14. Principal component analysis of the Thoroughbred (TB) racehorse ProteoMiner^{TM} processed (16hr + 2hr trypsin digestion) synovial fluid proteome categorised by (A) macroscopic OA grade (n=47), (B) microscopic OA grade (n=45) and (C) synovitis grade (n=53) using LC-MS/MS.

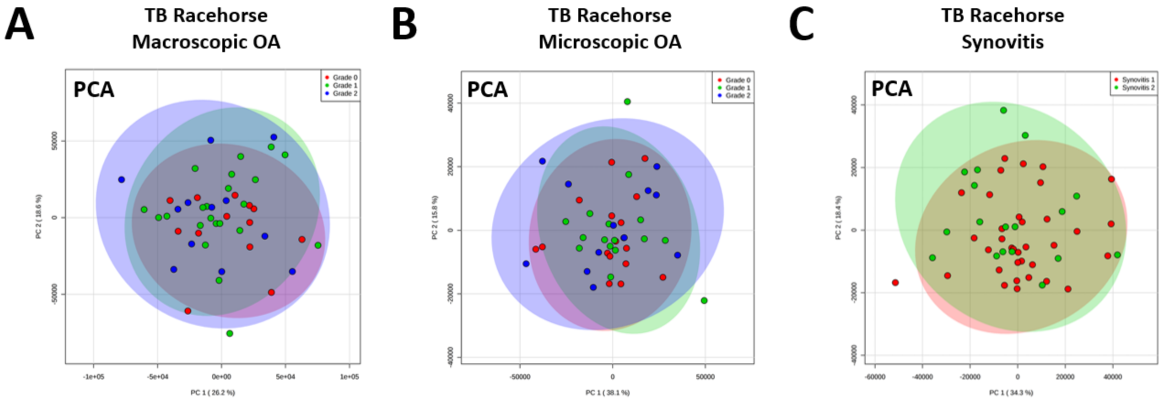

Supplement: Supplementary file 15 — Figure S14. Principal component analysis of the Thoroughbred (TB) racehorse ProteoMiner™ processed (16 h + 2 h trypsin digestion) synovial fluid proteome categorised by (A) macroscopic OA grade (n = 47), (B) microscopic OA grade (n = 45) and (C) synovitis grade (n = 53) using LC–MS/MS. [file EVJ-57-1204-s003.pdf]
