# Supplementary material for: Metabolomic and proteomic stratification of equine osteoarthritis
Source: Equine Vet J. 2025 Feb 19;57(5):1204–18. doi: 10.1111/evj.14490 (PMC12326899; doi:10.1111/evj.14490)

Equine Veterinary Journal BEVA

Figure S15. Principal component analysis (PCA) of equine synovial fluid semi-tryptic peptide profiles categorised according to (A) microscopic osteoarthritis (n=62) and (B) macroscopic osteoarthritis (n=59) grading for the mixed breeds sample set and (C) microscopic osteoarthritis (n=46), (D) macroscopic osteoarthritis (n=46) and (E) synovitis (n=53) grading for the Thoroughbred (TB) racehorse sample set.

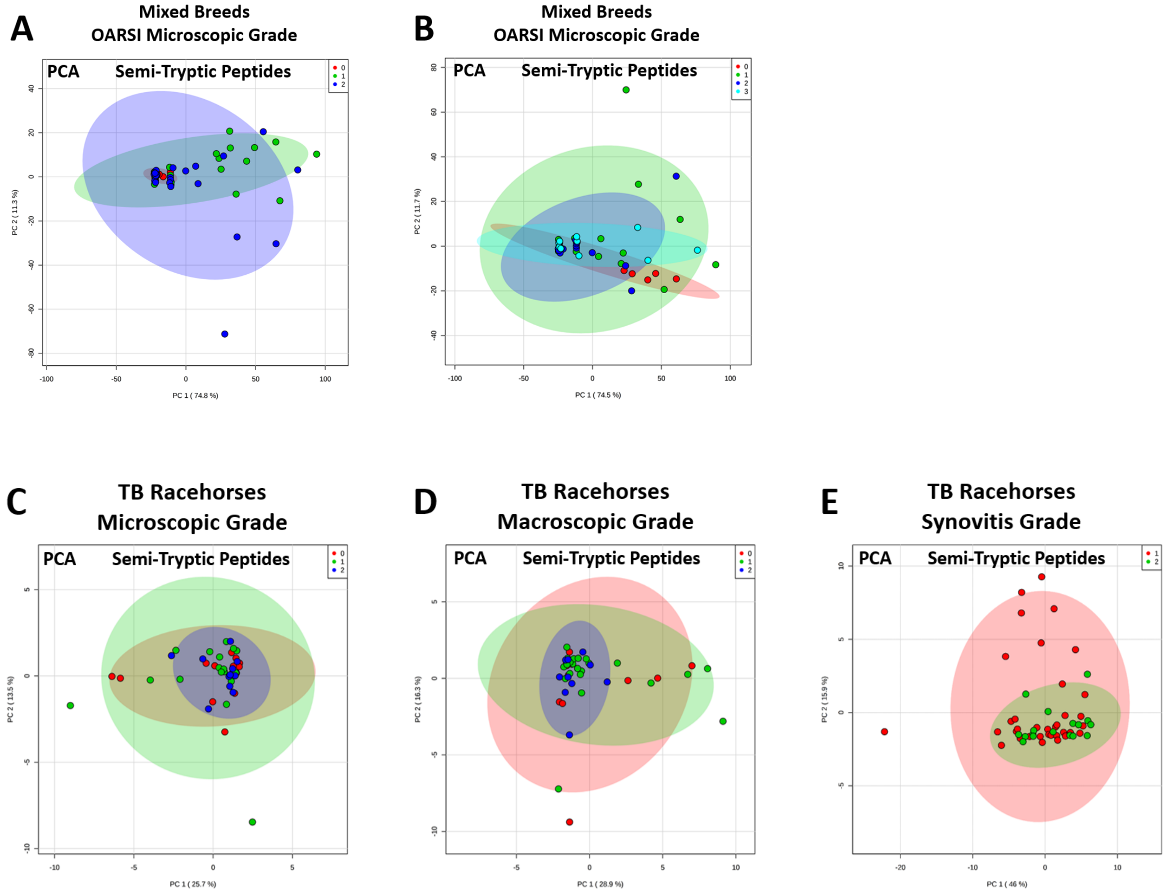

Supplement: Supplementary file 16 — Figure S15. Principal component analysis (PCA) of equine synovial fluid semi‐tryptic peptide profiles categorised according to (A) microscopic osteoarthritis (n = 62) and (B) macroscopic osteoarthritis (n = 59) grading for the mixed breeds sample set and (C) microscopic osteoarthritis (n = 46), (D) macroscopic osteoarthritis (n = 46) and (E) synovitis (n = 53) grading for the Thoroughbred (TB) racehorse sample set. [file EVJ-57-1204-s008.pdf]
