# Supplementary material for: Metabolomic and proteomic stratification of equine osteoarthritis
Source: Equine Vet J. 2025 Feb 19;57(5):1204–18. doi: 10.1111/evj.14490 (PMC12326899; doi:10.1111/evj.14490)
Supplement: Supplementary file 17 — Table S1. Macroscopic osteoarthritis scoring of distal metacarpal III for the mixed breeds sample set. [file EVJ-57-1204-s021.pdf]

Equine Veterinary Journal BEVA

Table S1. Macroscopic osteoarthritis scoring of distal metacarpal III for the mixed breeds sample set.

Horse	Scorer 1				Scorer 2				Scorer 3				Average TOTAL	Macroscopic OA Grade
	Wear Lines (0-3)	Erosions (0-3)	Palmar Arthrosis (0-3)	Total	Wear Lines (0-3)	Erosions (0-3)	Palmar Arthrosis (0-3)	Total	Wear Lines (0-3)	Erosions (0-3)	Palmar Arthrosis (0-3)	Total		
**1**	Not Scored				Not Scored									
**2**	0	2	0	**2**	1	2	0	**3**					3	**3**
**3**	0	0	0	**0**	0	0	0	**0**					0	**0**
**4**	Not Scored				Not Scored									
**5**	Not Scored				Not Scored									
**6**	Not Scored				Not Scored									
**7**	0	1	0	**1**	0	0	0	**0**					1	**1**
**8**	1	0	2	**3**	2	1	1	**4**					4	**3**
**9**	0	1	0	**1**	0	0	0	**0**					1	**1**
**10**	0	0	0	**0**	0	1	1	**2**	0	1	0	**1**	2	**2**
**11**	0	1	0	**1**	1	1	0	**2**					2	**2**
**12**	0	0	1	**1**	1	1	0	**2**					2	**2**
**13**	1	2	1	**4**	1	1	3	**5**					5	**3**
**14**	0	0	0	**0**	1	0	1	**2**	1	0	0	**1**	2	**2**
**15**	Not Scored				Not Scored									
**16**	0	0	1	**1**	1	0	2	**3**	1	0	0	**1**	1	**1**
**17**	Not Scored				Not Scored									
**18**	0	0	0	**0**	0	0	0	**0**					0	**0**
**19**	0	2	2	**4**	0	0	0	**0**	0	0	0	**0**	0	**0**
**20**	0	0	0	**0**	0	0	0	**0**					0	**0**
**21**	0	2	0	**2**	1	1	0	**2**					2	**2**
**22**	0	0	0	**0**	0	0	0	**0**					0	**0**
**23**	0	1	0	**1**	0	1	0	**1**					1	**1**
**24**	0	1	0	**1**	0	1	0	**1**					1	**1**
**25**	0	1	0	**1**	0	1	0	**1**					1	**1**
**26**	0	1	0	**1**	0	0	1	**1**					1	**1**
**27**	1	2	0	**3**	0	1	0	**1**	0	1	0	**1**	1	**1**
**28**	2	0	0	**2**	1	0	0	**1**					2	**2**
**29**	0	1	1	**2**	0	2	0	**2**					2	**2**
**30**	2	2	0	**4**	3	2	1	**6**	3	2	1	**6**	6	**3**

**31**	0	2	0	**2**	0	2	0	**2**					2	**2**
**32**	3	1	0	**4**	2	1	0	**3**					4	**3**
**33**	1	1	0	**2**	0	0	1	**1**					2	**2**
**34**	1	1	0	**2**	0	1	0	**1**					2	**2**
**35**	2	2	0	**4**	3	2	2	**7**	2	1	2	**5**	5	**3**
**36**	0	0	1	**1**	0	0	1	**1**					1	**1**
**37**	0	0	0	**0**	0	0	1	**1**					1	**1**
**38**	0	1	0	**1**	0	2	0	**2**					2	**2**
**39**	0	2	1	**3**	1	2	1	**4**					4	**3**
**40**	0	1	0	**1**	0	2	0	**2**					2	**2**
**41**	0	2	0	**2**	0	1	0	**1**					2	**2**
**42**	Not Scored				Not Scored									
**43**	0	1	0	**1**	0	0	1	**1**					1	**1**
**44**	0	0	0	**0**	0	1	0	**1**					1	**1**
**45**	1	0	0	**1**	0	0	0	**0**					1	**1**
**46**	0	0	0	**0**	0	0	0	**0**					0	**0**
**47**	0	0	1	**1**	1	2	0	**3**	0	0	1	**1**	1	**1**
**48**	2	2	0	**4**	2	3	0	**5**					5	**3**
**49**	1	2	0	**3**	0	2	0	**2**					3	**3**
**50**	0	0	0	**0**	0	1	0	**1**					1	**1**
**51**	1	2	0	**3**	0	2	0	**2**					3	**3**
**52**	0	0	0	**0**	0	0	0	**0**					0	**0**
**53**	0	2	0	**2**	0	1	0	**1**					2	**2**
**54**	0	0	0	**0**	0	0	0	**0**					0	**0**
**55**	0	0	0	**0**	0	1	0	**1**					1	**1**
**56**	1	0	0	**1**	0	1	0	**1**					1	**1**
**57**	0	0	0	**0**	0	0	0	**0**					0	**0**
**58**	0	0	1	**1**	0	2	0	**2**					2	**2**
**59**	0	0	0	**0**	0	1	0	**1**					1	**1**
**60**	0	1	0	**1**	0	1	0	**1**					1	**1**
**61**	0	0	0	**0**	0	1	0	**1**					1	**1**
**62**	0	0	0	**0**	0	1	0	**1**					1	**1**
**63**	0	0	0	**0**	0	0	0	**0**					0	**0**
**64**	0	0	0	**0**	0	1	0	**1**					1	**1**
**65**	0	2	0	**2**	0	1	0	**1**					2	**2**
**66**	0	1	0	**1**	0	1	0	**1**					1	**1**
**67**	0	0	0	**0**	0	0	0	**0**					0	**0**
**68**	1	2	0	**3**	1	1	1	**3**					3	**3**

**69**	1	0	0	**1**	0	1	0	**1**					1	**1**
**70**	0	1	0	**1**	0	2	0	**2**					2	**2**
**71**	3	0	0	**3**	3	0	0	**3**					3	**3**
**72**	0	0	2	**2**	1	1	2	**4**	0	0	2	**2**	2	**2**
**73**	0	0	0	**0**	0	1	0	**1**					1	**1**
**132**	0	0	2	**2**	0	0	0	**0**	0	0	0	**0**	0	**0**
**133**	0	0	0	**0**	0	0	0	**0**					0	**0**
**134**	0	2	0	**2**	0	1	0	**1**					2	**2**
**135**	0	2	1	**3**	0	1	0	**1**	0	1	0	**1**	1	**1**
**136**	1	0	1	**2**	1	1	0	**2**					2	**2**
**137**	0	0	1	**1**	1	2	1	**4**	1	2	0	**3**	4	**3**
**138**	0	0	0	**0**	0	0	0	**0**					0	**0**
**139**	3	0	1	**4**	3	0	0	**3**					4	**3**
**140**	1	1	0	**2**	0	1	0	**1**					2	**2**
**141**	0	0	1	**1**	0	0	0	**0**					1	**1**
