# Supplementary material for: Metabolomic and proteomic stratification of equine osteoarthritis
Source: Equine Vet J. 2025 Feb 19;57(5):1204–18. doi: 10.1111/evj.14490 (PMC12326899; doi:10.1111/evj.14490)
Supplement: Supplementary file 18 — Table S2. Microscopic osteoarthritis scoring of distal metacarpal III for the mixed breeds sample set. [file EVJ-57-1204-s019.pdf]

Equine Veterinary Journal BEVA

Table S2. Microscopic osteoarthritis scoring of distal metacarpal III for the mixed breeds sample set.

	Scorer 1						Scorer 2						Scorer 3							
Horse	Fissuring (0-4)	Focal Cell Loss (0-4)	Chond. Nec. (0-4)	Chon. Form. (0-4)	Saff O Stain (0-4)	Total	Fissuring (0-4)	Focal Cell Loss (0-4)	Chond. Nec. (0-4)	Chon. Form. (0-4)	Saff O Stain (0-4)	Total	Fissuring (0-4)	Focal Cell Loss (0-4)	Chond. Nec. (0-4)	Chon. Form. (0-4)	Saff O Stain (0-4)	Total	Average TOTAL	Microscopic OA Grade
1	0	1	1	0	0	2	1	1	0	0	0	2							2	1
2	2	1	0	1	0	4	2	0	1	1	0	4							4	2
3	0	1	0	0	0	1	0	0	0	0	0	0							1	0
4	0	0	1	0	0	1	0	1	1	0	0	2							2	1
5	1	1	0	0	0	2	1	1	0	1	0	3							3	1
6	0	0	0	0	0	0	0	2	1	0	0	3	0	0	0	0	0	0	0	0
7	1	1	0	0	0	2	1	0	1	0	1	3							3	1
8	1	0	1	1	0	3	2	1	1	1	0	5	2	1	0	1	0	4	5	2
9	1	0	0	0	1	2	0	2	2	0	1	5	0	1	0	0	0	1	2	1
10	0	0	0	1	0	1	1	1	0	0	0	2							2	1
11	1	2	0	0	0	3	1	0	0	0	0	1	1	0	0	0	0	1	1	0
12	2	2	1	0	0	5	2	1	2	0	0	5							5	2
13	1	1	0	0	0	2	1	1	0	0	0	2							2	1
14	1	1	1	2	0	5	1	3	0	1	0	5							5	2
15	1	1	0	0	0	2	1	1	0	0	0	2							2	1
16	0	2	0	1	0	3	0	1	0	1	0	2							3	1
17	Not Scored						Not Scored													
18	0	0	0	0	0	0	0	1	0	0	0	1							1	0
19	1	2	0	1	0	4	0	1	0	0	0	1	1	0	0	0	0	1	1	0
20	0	2	0	0	0	2	0	2	0	0	0	2							2	1
21	2	0	1	2	1	6	3	1	1	1	2	8	1	3	0	1	2	7	8	2
22	0	1	0	0	0	1	0	0	1	0	0	1							1	0
23	1	0	2	0	0	3	0	0	0	0	0	0	2	1	0	0	0	3	3	1
24	0	0	0	1	0	1	1	0	0	1	0	2							2	1
25	0	1	1	0	0	2	0	1	1	1	0	3							3	1
26	0	1	0	0	0	1	0	1	1	0	0	2							2	1
27	0	0	0	0	0	0	0	0	0	0	0	0							0	0
28	1	0	0	1	0	2	1	3	0	0	0	4	1	2	1	0	0	4	4	2
29	1	1	0	0	0	2	0	0	0	1	0	1							2	1
30	2	1	1	1	0	5	1	0	0	2	0	3	0	1	0	3	0	4	5	2

**31**	2	1	1	1	4	**9**	1	0	1	1	0	**3**	1	2	0	0	0	**3**	**3**	**1**
**32**	0	1	1	1	0	**3**	1	0	0	2	0	**3**							**3**	**1**
**33**	2	2	1	1	1	**7**	2	1	1	1	0	**5**	2	1	2	1	0	**6**	**7**	**2**
**34**	0	1	2	1	0	**4**	0	2	1	1	0	**4**							**4**	**2**
**35**	1	0	1	2	1	**5**	1	1	1	2	1	**6**							**6**	**2**
**36**	1	1	0	1	0	**3**	1	0	0	0	1	**2**							**3**	**1**
**37**	0	1	1	1	1	**4**	0	2	1	0	0	**3**							**4**	**2**
**38**	1	0	1	1	0	**3**	1	1	2	1	0	**5**	2	1	2	1	0	**6**	**6**	**2**
**39**	0	1	0	1	2	**4**	0	0	1	0	1	**2**	0	1	0	0	2	**3**	**4**	**2**
**40**	1	0	1	2	2	**6**	1	0	0	1	1	**3**	2	0	1	2	2	**7**	**7**	**2**
**41**	2	1	1	2	0	**6**	1	2	2	3	0	**8**	2	2	0	1	0	**5**	**6**	**2**
**42**	0	2	1	0	1	**4**	0	1	1	0	0	**2**	0	2	0	1	1	**4**	**4**	**2**
**43**	1	0	1	1	0	**3**	1	0	1	0	0	**2**							**3**	**1**
**44**	0	1	1	2	1	**5**	1	0	1	1	1	**4**							**5**	**2**
**45**	0	1	1	0	0	**2**	0	1	1	0	0	**2**							**2**	**1**
**46**	0	0	0	1	0	**1**	0	1	1	0	0	**2**							**2**	**1**
**47**	0	1	0	1	1	**3**	1	0	0	0	1	**2**							**3**	**1**
**48**	0	1	1	0	0	**2**	0	1	1	2	0	**4**	1	1	0	0	0	**2**	**2**	**1**
**49**	1	1	0	1	1	**4**	0	0	0	1	0	**1**	0	1	1	1	1	**4**	**4**	**2**
**50**	1	1	2	0	0	**4**	2	0	1	0	0	**3**							**4**	**2**
**51**	0	1	1	0	1	**3**	1	0	1	2	1	**5**	0	0	0	2	0	**2**	**3**	**1**
**52**	Not Scored						Not Scored													
**53**	1	0	0	2	1	**4**	2	0	0	1	0	**3**							**4**	**2**
**54**	0	1	1	0	0	**2**	0	2	0	0	0	**2**							**2**	**1**
**55**	0	1	1	0	1	**3**	0	0	0	0	0	**0**	0	1	0	0	1	**2**	**3**	**1**
**56**	1	1	0	0	2	**4**	0	1	0	0	1	**2**	1	1	0	0	2	**4**	**4**	**2**
**57**	0	1	0	0	1	**2**	0	2	1	0	1	**4**	1	1	0	0	0	**2**	**2**	**1**
**58**	0	1	0	2	0	**3**	0	1	2	0	0	**3**							**3**	**1**
**59**	0	0	0	0	0	**0**	0	1	0	1	1	**3**	1	1	0	1	1	**4**	**4**	**2**
**60**	1	0	0	1	0	**2**	2	0	0	2	0	**4**	0	2	0	2	0	**4**	**4**	**2**
**61**	0	1	1	1	0	**3**	0	0	1	0	2	**3**							**3**	**1**
**62**	0	1	0	0	1	**2**	1	1	1	0	1	**4**	0	1	0	1	0	**2**	**2**	**1**
**63**	0	2	1	1	0	**4**	0	1	1	0	2	**4**							**4**	**2**
**64**	1	1	0	2	1	**5**	0	1	0	1	0	**2**	1	1	0	0	0	**2**	**2**	**1**
**65**	1	2	0	1	0	**4**	0	1	0	1	0	**2**	0	1	0	2	0	**3**	**4**	**2**
**66**	1	2	1	0	1	**5**	1	0	0	1	0	**2**	0	2	0	0	0	**2**	**2**	**1**
**67**	0	1	1	1	0	**3**	0	0	0	1	1	**2**							**3**	**1**
**68**	1	1	0	0	0	**2**	2	0	0	0	0	**2**							**2**	**1**

**69**	2	0	1	1	0	**4**	1	0	1	1	0	**3**							**4**	**2**
**70**	3	1	1	1	2	**8**	2	0	1	1	2	**6**	3	2	0	0	2	**7**	**8**	**2**
**71**	2	2	1	1	0	**6**	1	1	0	1	0	**3**	1	2	0	3	0	**6**	**6**	**2**
**72**	Not Scored						Not Scored													
**73**	2	1	1	0	0	**4**	0	0	0	0	0	**0**	1	0	0	0	0	**1**	**1**	**0**
**132**	Not Scored						Not Scored													
**133**	Not Scored						Not Scored													
**134**	Not Scored						Not Scored													
**135**	Not Scored						Not Scored													
**136**	Not Scored						Not Scored													
**137**	Not Scored						Not Scored													
**138**	Not Scored						Not Scored													
**139**	Not Scored						Not Scored													
**140**	Not Scored						Not Scored													
**141**	Not Scored						Not Scored													
