# Supplementary material for: Metabolomic and proteomic stratification of equine osteoarthritis
Source: Equine Vet J. 2025 Feb 19;57(5):1204–18. doi: 10.1111/evj.14490 (PMC12326899; doi:10.1111/evj.14490)
Supplement: Supplementary file 19 — Table S3. Macroscopic osteoarthritis scoring of distal metacarpal III or metatarsal III for the Thoroughbred racehorse sample set. [file EVJ-57-1204-s002.pdf]

Equine Veterinary Journal BEVA

Table S3. Macroscopic osteoarthritis scoring of distal metacarpal III or metatarsal III for the Thoroughbred racehorse sample set.

		Scorer 1					Scorer 2					Scorer 3						
**Horse**	**Joint**	**POD (0-3)**	**Wear lines (0-2)**	**Cart. loss (0-3)**	**Linear fissures (0-3)**	**TOTAL**	**POD (0-3)**	**Wear lines (0-2)**	**Cart. loss (0-3)**	**Linear fissures (0-3)**	**TOTAL**	**POD (0-3)**	**Wear lines (0-2)**	**Cart. loss (0-3)**	**Linear fissures (0-3)**	**TOTAL**	**Average TOTAL**	**Macroscopic OA Grade**
74	MCP	Not Scored					Not Scored											
75	MCP	2	0	3	0	5	1	0	1	0	2	2	0	2	0	4	5	**2**
76	MCP	0	2	2	1	5	2	2	1	0	5						5	**2**
77	MCP	3	2	3	1	9	3	2	3	0	8						9	**2**
78	MCP	0	2	0	2	4	0	1	0	0	1	0	1	0	1	2	2	**0**
79	MCP	0	1	0	2	3	0	1	1	0	2						3	**1**
80	MCP	3	2	1	0	6	3	2	1	0	6						6	**2**
81	MCP	Not Scored					Not Scored											
82	MCP	2	1	0	0	3	2	1	1	0	4						4	**1**
83	MCP	1	2	1	0	4	1	2	1	0	4						4	**1**
84	MCP	0	0	3	1	4	0	0	1	1	2	0	1	1	1	3	4	**1**
85	MCP	0	2	3	0	5	0	2	1	0	3	0	2	1	0	3	3	**1**
86	MCP	2	2	3	0	7	2	1	1	0	4	1	1	1	0	3	4	**1**
87	MCP	3	2	3	0	8	3	2	1	0	6	2	1	2	0	5	6	**2**
88	MCP	1	0	0	0	1	1	0	0	2	3	1	1	0	1	3	3	**1**
89	MCP	3	2	3	0	8	3	2	1	0	6	3	2	3	0	8	8	**2**
90	MCP	1	0	3	0	4	1	0	1	0	2	1	1	1	0	3	4	**1**
91	MCP	1	1	2	0	4	1	1	1	0	3						4	**1**
92	MCP	3	2	3	0	8	1	1	1	0	3						6	**2**
93	MCP	2	0	3	0	5	2	0	2	1	5						5	**2**
94	MCP	0	0	2	0	2	1	1	1	0	3						3	**1**
95	MCP	2	1	3	3	9	1	1	1	3	6	2	1	2	1	6	6	**2**
96	MCP	2	0	3	0	5	2	0	1	1	4						5	**2**
97	MCP	2	2	1	0	5	2	1	0	0	3	1	2	1	0	4	5	**2**
98	MCP	0	0	0	1	1	0	0	0	1	1						1	**0**
99	MCP	0	0	0	0	0	0	1	0	1	2	0	0	0	0	0	0	**0**
100	MCP	0	1	0	2	3	1	1	0	1	3						3	**1**
101	MCP	1	0	0	0	1	0	0	1	1	2						2	**0**

102	MCP	1	1	2	0	4	1	1	2	1	5						5	**2**
103	MCP	0	0	0	1	1	0	0	1	1	2						2	**0**
104	MCP	Not Scored					Not Scored											
105	MTP	Not Scored					Not Scored											
106	MTP	0	0	2	2	4	0	0	1	1	2	0	0	0	1	1	2	**0**
107	MTP	Not Scored					Not Scored											
108	MTP	0	0	1	2	3	1	0	1	1	3						3	**1**
109	MTP	2	0	1	2	5	2	0	1	2	5						5	**2**
110	MTP	3	2	3	0	8	0	0	0	1	1	0	1	1	0	2	2	**0**
111	MTP	0	2	2	0	4	1	1	0	1	3						4	**1**
112	MTP	Not Scored					Not Scored											
113	MTP	0	1	3	1	5	1	1	0	1	3	0	1	0	2	3	3	**1**
114	MTP	1	0	1	0	2	1	0	1	1	3						3	**1**
115	MTP	0	1	0	3	4	1	1	0	2	4						4	**1**
116	MTP	0	0	0	3	3	0	0	0	2	2						3	**1**
117	MTP	0	0	0	3	3	0	1	0	1	2						3	**1**
118	MTP	0	0	0	2	2	1	0	0	2	3						3	**1**
119	MTP	0	0	1	2	3	0	0	0	2	2						3	**1**
120	MTP	1	1	0	0	2	0	1	0	2	3						3	**1**
121	MTP	0	0	0	1	1	0	0	0	1	1						1	**0**
122	MTP	0	0	0	3	3	0	0	0	1	1						2	**0**
123	MTP	1	1	0	0	2	1	0	0	2	3						3	**1**
124	MTP	Not Scored					Not Scored											
125	MTP	2	0	0	2	4	1	0	0	1	2						3	**1**
126	MTP	Not Scored					Not Scored											
127	MTP	0	0	0	1	1	0	0	0	2	2						2	**0**
128	MTP	0	0	0	2	2	0	0	0	2	2						2	**0**
129	MTP	1	0	0	1	2	1	0	0	2	3						3	**1**
130	MTP	0	0	0	2	2	0	0	0	1	1						2	**0**
131	MCP	0	1	3	1	5	1	1	1	0	3						4	**1**
