# Supplementary material for: Metabolomic and proteomic stratification of equine osteoarthritis
Source: Equine Vet J. 2025 Feb 19;57(5):1204–18. doi: 10.1111/evj.14490 (PMC12326899; doi:10.1111/evj.14490)
Supplement: Supplementary file 20 — Table S4. Microscopic osteoarthritis scoring of distal metacarpal III or metatarsal III for the Thoroughbred racehorse sample set. [file EVJ-57-1204-s010.pdf]

Equine Veterinary Journal BEVA

Table S4. Microscopic osteoarthritis scoring of distal metacarpal III or metatarsal III for the Thoroughbred racehorse sample set.

Horse	Joint	Structure (0-10)	Cell Density (0-4)	Cell Cloning (0-4)	Staining (0-4)	Tidemark (0-3)	TOTAL	Microscopic OA Grade
74	MCP	Not Scored						
75	MCP	1	0	2	2	3	8	**1**
76	MCP	8	1	4	4	3	20	**2**
77	MCP	1	0	1	1	2	5	**1**
78	MCP	2	0	0	1	1	4	**0**
79	MCP	1	1	1	0	0	3	**0**
80	MCP	Not Scored						
81	MCP	Not Scored						
82	MCP	8	4	4	2	1	19	**2**
83	MCP	6	2	3	4	1	16	**2**
84	MCP	0	1	1	0	0	2	**0**
85	MCP	1	1	1	2	1	6	**1**
86	MCP	1	2	2	1	2	8	**1**
87	MCP	0	0	0	0	0	0	**0**
88	MCP	3	0	2	2	3	10	**1**
89	MCP	2	1	1	3	2	9	**1**
90	MCP	7	1	0	3	3	14	**2**
91	MCP	7	1	1	4	3	16	**2**
92	MCP	10	1	0	4	3	18	**2**
93	MCP	4	2	0	2	3	11	**2**
94	MCP	2	1	2	1	3	9	**1**
95	MCP	10	1	2	2	3	18	**2**
96	MCP	Not Scored						
97	MCP	1	0	1	0	1	3	**0**
98	MCP	0	0	1	0	0	1	**0**
99	MCP	2	1	0	0	0	3	**0**
100	MCP	Not Scored						
101	MCP	3	0	0	2	2	7	**1**
102	MCP	10	2	0	3	3	18	**2**
103	MCP	1	0	0	1	2	4	**0**
104	MCP	2	0	2	0	0	4	**0**
105	MTP	Not Scored						
106	MTP	1	0	0	1	0	2	**0**
107	MTP	2	0	1	2	2	7	**1**
108	MTP	5	0	0	2	3	10	**1**
109	MTP	2	1	1	1	0	5	**1**
110	MTP	4	2	0	4	2	12	**2**
111	MTP	5	1	1	3	2	12	**2**
112	MTP	Not Scored						
113	MTP	2	0	1	1	1	5	**1**
114	MTP	2	1	3	4	2	12	**2**
115	MTP	3	3	4	1	1	12	**2**
116	MTP	2	0	1	0	0	3	**0**
117	MTP	1	1	1	2	2	7	**1**
118	MTP	3	0	1	3	1	8	**1**
119	MTP	1	0	0	1	0	2	**0**

120	MTP	1	0	1	1	0	3	**0**
121	MTP	1	1	1	2	3	8	**1**
122	MTP	1	1	1	0	0	3	**0**
123	MTP	1	0	0	3	3	7	**1**
124	MTP	Not Scored						
125	MTP	2	1	1	2	0	6	**1**
126	MTP	2	0	0	1	2	5	**1**
127	MTP	2	1	1	2	2	8	**1**
128	MTP	1	0	1	1	3	6	**1**
129	MTP	1	0	0	1	2	4	**0**
130	MTP	1	1	1	1	0	4	**0**
131	MCP	0	1	1	1	0	3	**0**
