# Supplementary material for: Metabolomic and proteomic stratification of equine osteoarthritis
Source: Equine Vet J. 2025 Feb 19;57(5):1204–18. doi: 10.1111/evj.14490 (PMC12326899; doi:10.1111/evj.14490)
Supplement: Supplementary file 21 — Table S5. Synovitis scoring of distal metacarpal III or metatarsal III for the Thoroughbred racehorse sample set. [file EVJ-57-1204-s023.pdf]

Equine Veterinary Journal BEVA

Table S5. Synovitis scoring of distal metacarpal III or metatarsal III for the Thoroughbred racehorse sample set.

		Lining Cell Layer (0-3)				Resident Cells (0-3)				Inflammatory infiltrate (0-3)					
Horse	Joint	1	2	3	Average	1	2	3	Average	1	2	3	Average	Total	Synovitis Grade
74	MCP	1	1	0	**0.7**	2	2	0	**1.3**	2	2	1	**1.7**	**4**	**1**
75	MCP	1	1	1	**1.0**	1	2	1	**1.3**	0	0	1	**0.3**	**3**	**1**
76	MCP	2	2	1	**1.7**	1	1	1	**1.0**	1	2	1	**1.3**	**4**	**1**
77	MCP	2	1	3	**2.0**	2	2	1	**1.7**	1	1	1	**1.0**	**5**	**2**
78	MCP	2	1	1	**1.3**	1	1	1	**1.0**	1	1	1	**1.0**	**3**	**1**
79	MCP	1	2	2	**1.7**	1	1	1	**1.0**	1	1	1	**1.0**	**4**	**1**
80	MCP	1	2	2	**1.7**	1	1	1	**1.0**	1	1	1	**1.0**	**4**	**1**
81	MCP	0	1	1	**0.7**	1	2	2	**1.7**	0	3	3	**2.0**	**4**	**1**
82	MCP	1	1	3	**1.7**	1	2	2	**1.7**	2	2	3	**2.3**	**6**	**2**
83	MCP	2	1	2	**1.7**	1	1	1	**1.0**	1	1	1	**1.0**	**4**	**1**
84	MCP	1	1	2	**1.3**	1	1	1	**1.0**	1	1	1	**1.0**	**3**	**1**
85	MCP	2	2	2	**2.0**	2	1	1	**1.3**	1	1	1	**1.0**	**4**	**1**
86	MCP	2	2	1	**1.7**	1	1	1	**1.0**	1	1	1	**1.0**	**4**	**1**
87	MCP	2	3	1	**2.0**	3	2	3	**2.7**	0	1	0	**0.3**	**5**	**2**
88	MCP	2	2	2	**2.0**	1	1	1	**1.0**	2	1	1	**1.3**	**4**	**1**
89	MCP	2	3	2	**2.3**	2	2	2	**2.0**	2	2	2	**2.0**	**6**	**2**
90	MCP	3	2	3	**2.7**	3	3	3	**3.0**	2	2	2	**2.0**	**8**	**2**
91	MCP	1	2	2	**1.7**	1	2	1	**1.3**	1	1	2	**1.3**	**4**	**1**
92	MCP	2	3	3	**2.7**	1	1	2	**1.3**	1	1	2	**1.3**	**5**	**2**
93	MCP	3	2	2	**2.3**	2	1	3	**2.0**	1	2	1	**1.3**	**6**	**2**
94	MCP	0	1	1	**0.7**	1	2	1	**1.3**	1	1	2	**1.3**	**3**	**1**
95	MCP	2	2	3	**2.3**	3	2	3	**2.7**	2	2	2	**2.0**	**7**	**2**
96	MCP	1	1	3	**1.7**	2	2	2	**2.0**	1	1	1	**1.0**	**5**	**2**
97	MCP	1	2	3	**2.0**	1	1	1	**1.0**	1	1	1	**1.0**	**4**	**1**
98	MCP	2	1	1	**1.3**	1	1	1	**1.0**	1	1	1	**1.0**	**3**	**1**
99	MCP	1	1	1	**1.0**	1	1	1	**1.0**	1	1	1	**1.0**	**3**	**1**
100	MCP	0	1	1	**0.7**	1	1	1	**1.0**	0	1	1	**0.7**	**2**	**1**
101	MCP	0	0	0	**0.0**	1	1	1	**1.0**	1	1	1	**1.0**	**2**	**1**
102	MCP	3	3	3	**3.0**	2	2	3	**2.3**	2	2	1	**1.7**	**7**	**2**
103	MCP	1	0	2	**1.0**	1	1	1	**1.0**	2	2	1	**1.7**	**4**	**1**
104	MCP	0	1	2	**1.0**	0	1	1	**0.7**	1	1	1	**1.0**	**3**	**1**
105	MTP	1	2	2	**1.7**	1	3	3	**2.3**	2	2	2	**2.0**	**6**	**2**
106	MTP	2	1	2	**1.7**	1	1	1	**1.0**	1	1	1	**1.0**	**4**	**1**
107	MTP	1	2	1	**1.3**	1	1	1	**1.0**	1	1	1	**1.0**	**3**	**1**
108	MTP	1	1	0	**0.7**	0	1	0	**0.3**	1	1	1	**1.0**	**2**	**1**
109	MTP	2	2	3	**2.3**	1	1	1	**1.0**	1	1	1	**1.0**	**4**	**1**
110	MTP	3	1	0	**1.3**	1	1	0	**0.7**	1	2	1	**1.3**	**3**	**1**
111	MTP	2	2	0	**1.3**	1	1	1	**1.0**	1	1	1	**1.0**	**3**	**1**
112	MTP	2	2	1	**1.7**	1	1	1	**1.0**	2	1	2	**1.7**	**4**	**1**
113	MTP	2	3	1	**2.0**	1	2	0	**1.0**	1	3	0	**1.3**	**4**	**1**
114	MTP	1	1	1	**1.0**	1	1	1	**1.0**	1	1	1	**1.0**	**3**	**1**
115	MTP	0	0	1	**0.3**	0	0	0	**0.0**	1	1	1	**1.0**	**1**	**0**
116	MTP	2	3	3	**2.7**	1	1	1	**1.0**	1	1	1	**1.0**	**5**	**2**
117	MTP	1	1	2	**1.3**	0	1	1	**0.7**	1	1	1	**1.0**	**3**	**1**
118	MTP	3	2	2	**2.3**	2	1	2	**1.7**	1	1	1	**1.0**	**5**	**2**

119	MTP	2	2	2	**2.0**	2	3	1	**2.0**	2	2	2	**2.0**	**6**	**2**
120	MTP	1	1	1	**1.0**	1	1	1	**1.0**	1	1	1	**1.0**	**3**	**1**
121	MTP	0	2	2	**1.3**	1	1	3	**1.7**	2	2	3	**2.3**	**5**	**2**
122	MTP	2	1	3	**2.0**	1	1	1	**1.0**	1	1	2	**1.3**	**4**	**1**
123	MTP	2	2	1	**1.7**	1	1	1	**1.0**	2	1	1	**1.3**	**4**	**1**
124	MTP	2	2	2	**2.0**	1	2	1	**1.3**	1	1	1	**1.0**	**4**	**1**
125	MTP	2	2	2	**2.0**	1	1	0	**0.7**	1	1	1	**1.0**	**4**	**1**
126	MTP	1	2	1	**1.3**	1	1	1	**1.0**	1	1	1	**1.0**	**3**	**1**
127	MTP	0	2	2	**1.3**	1	2	2	**1.7**	1	2	2	**1.7**	**5**	**2**
128	MTP	1	3	3	**2.3**	1	1	2	**1.3**	1	3	3	**2.3**	**6**	**2**
129	MTP	2	2	1	**1.7**	2	2	1	**1.7**	1	2	1	**1.3**	**5**	**2**
130	MTP	3	3	2	**2.7**	1	1	1	**1.0**	1	1	1	**1.0**	**5**	**2**
131	MCP	1	3	2	**2.0**	1	1	1	**1.0**	1	1	1	**1.0**	**4**	**1**
