# Supplementary material for: Metabolomic and proteomic stratification of equine osteoarthritis
Source: Equine Vet J. 2025 Feb 19;57(5):1204–18. doi: 10.1111/evj.14490 (PMC12326899; doi:10.1111/evj.14490)
Supplement: Supplementary file 22 — Table S6. Correlation of each variable (proteins and metabolites) to macroscopic OA score for the Thoroughbred racehorse synovial fluid integrated dataset. p < 0.05. [file EVJ-57-1204-s007.pdf]

Table S6. Correlation of each variable (proteins and metabolites) to macroscopic OA score for the Thoroughbred racehorse synovial fluid integrated dataset. $p < 0.05$.

Variable	Correlation	Permutation p value	Characterisation
P60708	-0.44	0	Actin, cytoplasmic 1
HMDB00001	0.37	0	τ-Methylhistidine
F6UL68	-0.45	0	Transthyretin
F6VCB4	-0.36	0	Histone H3
F6VS95	-0.48	0	Protein disulfide isomerase family A member 6
F6W3T1	-0.38	0	L-lactate dehydrogenase
F6YNM7	-0.36	0	Prolyl endopeptidase
F6YRC5	-0.39	0	IQ motif containing GTPase activating protein 1
F6YRE0	-0.36	0	Tubulin alpha chain
F6ZMJ5	-0.40	0	ATPase H^+ transporting V1 subunit E2
F7ABC9	-0.38	0	Fibulin-1
F7D8W6	-0.43	0	Guanylate binding protein 2
F7DNT0	-0.42	0	Tubulin alpha chain
F7DYV0	-0.43	0	Galectin
A2Q0Z0	-0.28	0.01	Elongation factor 1-alpha 1
Q28372	-0.35	0.01	Gelsolin
F6T0P6	-0.36	0.01	GC, vitamin D binding protein
F6UZI2	-0.35	0.01	Coagulation factor XIII A chain
F6V6L8	-0.36	0.01	IQ motif containing GTPase activating protein 2
F6YIU8	-0.39	0.01	Methylenetetrahydrofolate dehydrogenase, cyclohydrolase and formyltetrahydrofolate synthetase 1
F6YXS1	0.37	0.01	Receptor protein-tyrosine kinase
F6ZFH9	-0.39	0.01	Tyrosine 3-monooxygenase/tryptophan 5-monooxygenase activation protein gamma
F7AYC1	-0.31	0.01	Secreted phosphoprotein 1

F7BNQ2	-0.36	0.01	Complement component 4 binding protein alpha
F7BQD6	0.35	0.01	Complement C1s
F7CYG2	-0.38	0.01	Tyrosine 3-monooxygenase/tryptophan 5-monooxygenase activation protein eta
HMDB00062	0.35	0.02	Carnitine
HMDB00190	-0.33	0.02	Lactate
F6QXN5	-0.34	0.02	Transgelin
F6S0P5	-0.34	0.02	ADP ribosylation factor 4
F6S2C3	-0.33	0.02	Cathepsin Z
F6UN85	-0.40	0.02	Carboxylic ester hydrolase
F6Y0G5	0.42	0.02	Periostin
F6Y4J0	-0.31	0.02	ATPase H^+ transporting V1 subunit B2
F6Z8W0	-0.36	0.02	WD repeat domain 1
F7APS1	-0.27	0.02	Uncharacterised
F7BM69	-0.31	0.02	Uncharacterised
F7BTK9	-0.36	0.02	Family with sequence similarity 129 member A
F7DA17	-0.39	0.02	Transglutaminase 2
F7DEW5	-0.31	0.02	Coronin
F7DXM5	0.32	0.02	Uncharacterised
HMDB00161	0.29	0.03	L-Alanine
F6PKE1	-0.33	0.03	Uncharacterised
F6QKR7	-0.38	0.03	Myocilin
F6RCA8	-0.34	0.03	Peroxiredoxin 5
F6YV40	-0.31	0.03	Glyceraldehyde-3-phosphate dehydrogenase
F6YV53	-0.31	0.03	Actinin alpha 4
HMDB00122	0.30	0.04	D-Glucose
Q95182	-0.34	0.04	Major allergen Equ c 1
F6RMN2	-0.34	0.04	Drebrin like
F6SN52	-0.34	0.04	Eukaryotic translation initiation factor 5A

F6SP02	-0.33	0.04	Tyrosine 3-monooxygenase/tryptophan 5-monooxygenase activation protein theta
F6T201	-0.29	0.04	Chondroitin sulfate proteoglycan 4
F6UF75	-0.33	0.04	Protein S100
F6WWW7	-0.32	0.04	Capping actin protein of muscle Z-line alpha subunit 1
F6XEB4	-0.36	0.04	Tyrosine 3-monooxygenase/tryptophan 5-monooxygenase activation protein zeta
F7B320	-0.32	0.04	Dihydropyrimidinase like 2
H9GZU9	-0.30	0.04	Uncharacterised
