# Supplementary material for: Metabolomic and proteomic stratification of equine osteoarthritis
Source: Equine Vet J. 2025 Feb 19;57(5):1204–18. doi: 10.1111/evj.14490 (PMC12326899; doi:10.1111/evj.14490)
Supplement: Supplementary file 23 — Table S7. Correlation of each variable (proteins and metabolites) to macroscopic OA score for the mixed‐breed synovial fluid integrated dataset. p < 0.05. [file EVJ-57-1204-s011.pdf]

Equine Veterinary Journal BEVA

Table S7. Correlation of each variable (proteins and metabolites) to macroscopic OA score for the mixed breeds synovial fluid integrated dataset. $p < 0.05$.

Variable	Correlation	Permutation p value	Characterisation
H9GZS6	-0.44	0	Uncharacterised
F7BFT1	-0.43	0	Peroxiredoxin 2
F6Q4N3	-0.41	0	Neural EGFL like 2
F6WCB7	-0.40	0	Cytokine like 1
F6ZR63	-0.38	0	Uncharacterised
H9GZU9	-0.37	0	Uncharacterised
F6RRV1	-0.35	0	Fetuin B
P35747	0.33	0	Serum albumin
Q28369	0.34	0	Retinol-binding protein 4
F6X667	0.34	0	Vitamin K-dependent protein C
F7A1W7	0.49	0	Apolipoprotein C2
H9GZV0	-0.34	0.01	Uncharacterised
F6SP11	-0.32	0.01	Uncharacterised
F6ZRF6	-0.31	0.01	Serpin family A member 7
F6QF58	-0.31	0.01	60S ribosomal protein L6
H9GZQ9	-0.29	0.01	Uncharacterised
F7ASE1	0.32	0.01	Interleukin 1 receptor accessory protein
F6RM73	0.36	0.01	Apolipoprotein A-II
F7DRS2	-0.33	0.02	Serpin family A member 6
F6WLX9	-0.29	0.02	tRNA-splicing ligase RtcB homolog
F6XPL9	-0.27	0.02	Obg-like ATPase 1
F6XI92	-0.27	0.02	Bridging integrator 2
F6YV40	0.21	0.02	Glyceraldehyde-3-phosphate dehydrogenase
F7DBT2	0.27	0.02	Complement C1q C chain
F6PRI5	0.28	0.02	Carboxylic ester hydrolase

F6T0P6	0.32	0.02	GC, vitamin D binding protein
F6UCZ2	-0.24	0.03	Translin
HMDB00562	0.30	0.03	Creatinine
F6XWJ6	-0.32	0.04	RUN and FYVE domain containing 1
F6V1V8	-0.27	0.04	Cluster of differentiation 109
F7BA94	-0.20	0.04	40S ribosomal protein S3
F7AS57	0.24	0.04	Proteasome activator subunit 2
