# Supplementary material for: Metabolomic and proteomic stratification of equine osteoarthritis
Source: Equine Vet J. 2025 Feb 19;57(5):1204–18. doi: 10.1111/evj.14490 (PMC12326899; doi:10.1111/evj.14490)
Supplement: Supplementary file 24 — Table S8. BLAST analysis of amino acid sequences of uncharacterised proteins included within this study, identifying the characterised protein with the highest percentage amino acid sequence similarity for each uncharacterised protein. [file EVJ-57-1204-s009.pdf]

Equine Veterinary Journal
BEVA

Table S8. BLAST analysis of amino acid sequences of uncharacterised proteins included within this study, identifying the characterised protein with the highest percentage amino acid sequence similarity for each uncharacterised protein.

Accession Number of Uncharacterised Protein	Analysis Present In	Accession Number of Similar Protein	Similar Protein	Species	E-Value	Similarity (%)
F6PKE1	TB Racehorse Macroscopic OA Correlation	A0A340XC13	Inhibitor of carbonic anhydrase-like isoform X1	*Lipotes vexillifer*	0	77.2
F6SP11	Mixed Breeds Macroscopic OA Correlation	A0A0A1E4I0	Immunoglobulin lambda light chain variable region	*Equus caballus*	$8.10\ e^{-60}$	100.0
F6TED1	Mixed Breeds Native (Macroscopic OA)	A0A091E338	Immunoglobulin kappa chain V-III region MOPC 63	*Fukomys damarensis*	$2.20\ e^{-44}$	70.1
F6ZR63	Mixed Breeds Macroscopic OA Correlation	A0A383YWT8	Complement factor H-like isoform X1	*Balaenoptera acutorostrata scammoni*	0	68.6
F7APS1	TB Racehorse Macroscopic OA Correlation	Q862Z5	Cystatin-B	*Macaca fuscata fuscata*	$2.30\ e^{-57}$	87.8
F7BM69	TB Racehorse Macroscopic OA Correlation	A0A337SQC1	Immunoglobulin kappa variable 4-1	*Felis catus*	$1.50\ e^{-56}$	77.4
F7DXM5	TB Racehorse Macroscopic OA Correlation	B5BV10	Alpha-1-antitrypsin	*Equus caballus*	0	98.1
H9GZQ9	Mixed Breeds Macroscopic OA Correlation	Q95M34	Immunoglobulin gamma 1 heavy chain constant region	*Equus caballus*	0	99.7
H9GZS6	Mixed Breeds Native (Macroscopic OA)	Q95M34	Immunoglobulin gamma 1 heavy chain constant region	*Equus caballus*	$7.40\ e^{-173}$	70.1
	Mixed Breeds Macroscopic OA Correlation					
H9GZU9	Mixed Breeds Native (Macroscopic OA)	Q95M34	Immunoglobulin gamma 1 heavy chain constant region	*Equus caballus*	$1.10\ e^{-159}$	67.9
	TB Racehorse Macroscopic OA Correlation					
	Mixed Breeds Macroscopic OA Correlation					
H9GZV0	Mixed Breeds Macroscopic OA Correlation	L5JR68	Immunoglobulin epsilon chain C region	*Pteropus alecto*	0	65.0

Abbreviations: TB, Thoroughbred; OA, Osteoarthritis
